# Identification and validation of shared biomarkers and drug repurposing in psoriasis and Crohn’s disease: integrating bioinformatics, machine learning, and experimental approaches

**DOI:** 10.3389/fimmu.2025.1587705

**Published:** 2025-05-08

**Authors:** Xiaolong Li, Hui Cao, Mutian Niu, Qingbo Liu, Bin Liang, Junfeng Hou, Jian Tu, Jintao Gao

**Affiliations:** ^1^ School of Intelligent Medicine and Biotechnology, Guilin Medical University, Guilin, Guangxi, China; ^2^ Key Laboratory of Molecular Medical Engineering, Education Department of Guangxi Zhuang Autonomous Region, Guilin, Guangxi, China; ^3^ Department of Dermatology, The Second Affiliated Hospital of Guilin Medical University, Guilin, Guangxi, China; ^4^ Pharmacy school of Guilin Medical University, Guilin, China; ^5^ Guangxi Key Laboratory of Molecular Medicine in Liver Injury and Repair, the Affiliated Hospital of Guilin Medical University, Guilin, China

**Keywords:** psoriasis, Crohn’s disease, immune, single-cell sequencing analysis, machine learning, bioinformatics

## Abstract

**Background:**

Psoriasis and Crohn’s disease (CD) are chronic inflammatory diseases that involve complex immune-mediated mechanisms. Despite clinical overlap and shared genetic predispositions, the molecular pathways connecting these diseases remain incompletely understood. The present study seeks to identify shared biomarkers and therapeutic targets for psoriasis and CD.

**Methods:**

Differentially expressed genes (DEGs) were identified from publicly available transcriptomic datasets related to psoriasis and CD. Simultaneously, weighted gene co-expression network analysis (WGCNA) was performed to identify gene modules associated with the clinical traits of psoriasis and CD. Subsequently, biomarkers were prioritized from shared key genes by integrating protein-protein interaction (PPI) networks with machine learning models. Gene Set Enrichment Analysis (GSEA), along with Gene Ontology (GO) and KEGG pathway analyses, were performed to determine the biological significance of the identified genes. Immune infiltration analysis underscored the involvement of hub genes in immune regulation, while single-cell transcriptomic analysis revealed the cellular localization of these hub genes. Additional targeted molecular biology experiments validated the shared biomarkers. DSigDB predictions were employed to identify potential therapeutic compounds. Molecular docking simulations were performed to assess the binding affinity of the drugs to key target proteins. Finally, additional in vitro experiments were conducted to validate the therapeutic effects of the identified compounds.

**Results:**

The study identified KIF4A, DLGAP5, NCAPG, CCNB1, and CEP55 as key regulatory molecules and shared biomarkers for both diseases. GSEA and pathway analysis highlighted the importance of cell cycle regulation and immune response pathways in the comorbidities of psoriasis and CD. Immune infiltration analysis emphasized the role of hub genes in immune regulation. Furthermore, DSigDB predictions and molecular docking simulations indicated strong therapeutic potential for Etoposide, Lucanthone, and Piroxicam, with Etoposide showing the highest affinity for key targets. In cellular models, Etoposide demonstrated promising therapeutic effects by significantly downregulating the expression of psoriasis-related keratinocytes marker genes (KRT6, KRT16) and CD-related inflammatory cytokines (IL6, IL8, TNF-α), highlighting its potential in treating psoriasis and CD.

**Discussion:**

This study integrates bioinformatics, machine learning, and molecular validation to identify the shared molecular mechanisms of psoriasis and CD, uncovering novel biomarkers and potential combined therapeutic candidates. These findings provide valuable insights into potential treatment strategies for these diseases.

## 1 Introduction

Psoriasis is a complex and chronic immune-mediated polygenic hereditary skin disorder influenced by a wide array of internal and external factors, including genetic predispositions, environmental triggers, and immunological irregularities (1). It is characterized by excessive proliferation of keratinocytes, abnormal differentiation, epidermal thickening, and infiltration of distinct inflammatory cell subsets such as T cells, dendritic cells, and neutrophils. These immunological disturbances are driven by a dysregulated cytokine network, with pivotal roles played by interleukin (IL)-17, IL-23, and tumor necrosis factor-alpha (TNF-α), which perpetuate chronic inflammation and skin lesions (2). Recent advances in molecular biology have facilitated the development of targeted therapies, including biologics such as IL-17 and IL-23 inhibitors, which have revolutionized psoriasis management. These therapies not only alleviate clinical symptoms but also improve patients’ quality of life by targeting the underlying inflammatory pathways. However, despite significant advancements, the precise pathogenesis of psoriasis remains incompletely understood, particularly regarding its systemic effects and associations with comorbid conditions (3, 4). Emerging evidence highlights a robust association between psoriasis and systemic diseases such as metabolic syndrome, cardiovascular disorders, and autoimmune conditions. The heightened cardiovascular risk in psoriasis patients, for example, is hypothesized to stem from chronic systemic inflammation, endothelial dysfunction, and increased prevalence of traditional risk factors such as obesity and dyslipidemia (3, 4). Despite these observations, the precise biological mechanisms linking psoriasis with systemic diseases remain inadequately characterized. Further investigations are necessary to fully elucidate these pathways.

Crohn’s disease (CD), a highly debilitating chronic and relapsing inflammatory bowel disease (IBD), is characterized by persistent inflammation affecting various parts of the gastrointestinal (GI) tract (5). The incidence and prevalence of CD are rising globally, particularly in Western countries and newly industrialized nations, seriously affecting the quality of life of patients (6, 7). While CD predominantly involves the terminal ileum and colon, it is frequently associated with extraintestinal manifestations, such as iridocyclitis and erythema nodosum (5, 8). Its pathogenesis is now generally accepted to result from a complex interplay of genetic susceptibility, gut microbiota dysbiosis, environmental factors, and abnormal immune responses (6, 9). Therefore, the exact interplay between genetic, microbial, and immunological factors remains poorly understood, necessitating continued research.

Importantly, psoriasis and CD demonstrate a significant degree of genetic and pathogenic overlap, with shared susceptibility loci. Genome-wide association studies (GWAS) have revealed strong evidence of shared genetic underpinnings and a bidirectional relationship between psoriasis and CD (10, 11). Patients with psoriasis are significantly more likely to develop CD, and vice versa (12, 13). This phenomenon is thought to result from shared genetic predispositions, overlapping pathogenic pathways, and specific interactions between the immune system and microbiota (10, 14–17). The prevailing hypothesis is that individuals with genetic susceptibility may develop these diseases through the interplay of environmental and immune factors, with epigenetic mechanisms such as DNA methylation and histone modification also playing a crucial role (18). However, the underlying mechanisms linking psoriasis and CD remain inadequately characterized. Additionally, current treatments for psoriasis and CD, such as the biologic agent ixekizumab and ustekinumab (UST), often lead to complex adverse reactions (19, 20). Therefore, further research is essential to uncover these connections and pave the way for innovative diagnostic and therapeutic approaches. Drug repurposing, also known as drug repositioning, is a strategy that accelerates the therapeutic process by identifying new indications for existing drugs (21). Traditional drug development typically takes decades, whereas drug repurposing can significantly shorten this timeline and reduce costs. Despite the availability of various treatment options for immune-related diseases like psoriasis and CD, challenges such as side effects and drug resistance highlight the urgent need for new therapeutic approaches. In this context, drug repurposing represents an innovative strategy that may provide effective alternative options.

**Figure 1:**
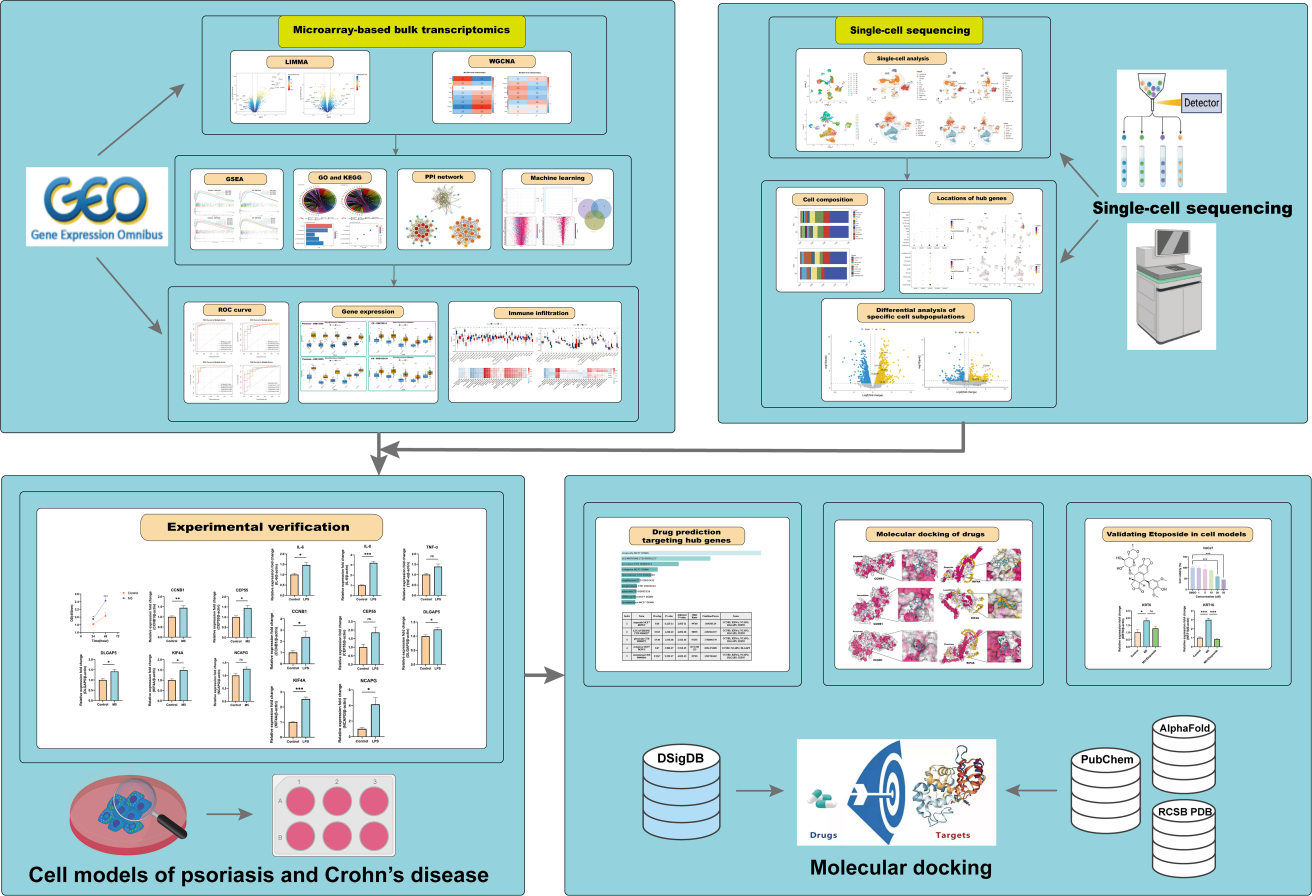
The comprehensive flowchart of this research.

This study integrates comprehensive bioinformatics approaches and machine learning to investigate the molecular mechanisms underlying the relationship between psoriasis and CD, as illustrated in the comprehensive flowchart (**Figure 1**). By identifying shared differentially expressed genes and critical biological pathways, KIF4A, DLGAP5, NCAPG, CCNB1, and CEP55 were recognized as novel co-biomarkers with potential diagnostic and therapeutic significance. Furthermore, molecular experiments and molecular docking analyses were employed to preliminarily identify potential therapeutic agents, which not only exhibit known safety profiles but also offer new perspectives for treatment strategies for both diseases.

## 2 Materials and methods

### 2.1 Bulk transcriptome data preprocessing

Microarray sequencing datasets related to psoriasis and CD were retrieved from the GEO database (https://www.ncbi.nlm.nih.gov/geo/). The search was conducted using the following Boolean search strategies: For psoriasis-related datasets: (“psoriasis”[MeSH Terms] OR psoriasis[All Fields]) AND “Homo sapiens”[orgn] AND (“gse”[Filter] AND “Expression profiling by array”[Filter]). For CD-related datasets: (“crohn disease”[MeSH Terms] OR Crohn’s disease[All Fields]) AND “Homo sapiens”[orgn] AND (“gse”[Filter] AND “Expression profiling by array”[Filter]). The entry type was restricted to “series”, the study type was limited to “expression profiling by array”, and the tissue source organism was restricted to “Homo sapiens”. Based on the strategy of selecting relatively large transcriptomic datasets, a total of four eligible gene expression datasets (GSE13355, GSE14905, GSE75214, and GSE102133) were selected. The GSE13355 dataset contains skin tissues from 58 patients with psoriasis and 64 normal healthy controls, while the GSE75214 dataset includes 67 CD samples and 11 control samples from ileal tissue. For validation datasets, GSE14905 contains 33 psoriasis samples and 21 normal control samples, and GSE102133 includes 65 CD samples and 12 normal control samples. Non-lesional samples were excluded to focus on examining differences between patient and normal control samples. These datasets are advantageous due to their relatively large sample sizes and have been widely cited in relevant literature, with their analysis results regarded as authoritative and reliable (22–25). Subsequently, data preprocessing was carried out as follows: The raw expression matrix was read using the “exprs()” function, and probe IDs were mapped to gene symbols. Background correction and quantile normalization were applied using the “normalizeBetweenArrays()” function from the “limma” package to adjust for technical variations between arrays. Additionally, log2 transformation was performed when significant numerical differences were observed, based on distribution checks. Data quality was visualized using box plots to ensure that no obvious outliers were present in the normalized data.

### 2.2 Single-cell transcriptome data

Single-cell transcriptomic data for psoriasis and CD were analyzed using datasets retrieved from the GEO database (https://www.ncbi.nlm.nih.gov/geo/). The psoriasis dataset (GSE162183) consisted of data from 3 psoriasis patients (lesional skin) and 3 healthy controls (normal skin). For CD, the GSE214695 dataset was utilized, focusing on colonic tissue from 6 CD patients and 6 healthy controls. Data processing and filtering were performed using the Seurat R package (v5.0.1) (26). The mitochondrial content of each cell was calculated with the Percentage_Feature_Set function. Quality control criteria for the GSE162183 dataset required each cell to express more than 300 genes, have a mitochondrial content of 5%–30%, and a total unique molecular identifier (UMI) count exceeding 1,000. For the GSE214695 dataset, the criteria included the expression of more than 300 genes, mitochondrial content of 5%–75%, and a total UMI count exceeding 1,000. After filtering, 15,592 and 23,591 high-quality cells were retained from the respective datasets for downstream analyses. Normalization was performed using the SCTransform function, with mitochondrial gene effects regressed out (vars.to.regress = “percent.mt”) to minimize technical biases. Dimensionality reduction was conducted using principal component analysis (PCA) and uniform manifold approximation and projection (UMAP), with the first 30 principal components (dims = 1:30) used for UMAP embedding. Cell clustering was achieved by constructing a shared nearest-neighbor (SNN) graph using the FindNeighbors function, followed by clustering with FindClusters.

### 2.3 Differential expression analysis

Differential expression analysis was performed on the GSE13355 (psoriasis) and GSE75214 (CD) datasets using the R package “limma”. Differentially expressed genes (DEGs) were identified with a significance threshold of *P*.adj.value < 0.05 and |log2 fold change (FC)| > 0.585. Shared DEGs between the psoriasis and CD datasets were identified using Venn diagrams generated with the online tool Evenn (https://jvenn.toulouse.inrae.fr/app/example.html).

### 2.4 Gene set enrichment analysis

Gene Set Enrichment Analysis (GSEA) was performed to explore the molecular pathways and mechanisms underlying the association between psoriasis and CD (27). To identify relevant pathways, we utilized the “clusterProfiler” package and the “h.all.v2024.1.Hs.symbols.gmt” gene set obtained from the Molecular Signatures Database (MSigDB) (28). Pathways significantly enriched in both psoriasis and CD were reported. Enriched gene sets with a nominal p-value < 0.05, |Normalized Enrichment Score (NES)| > 1, and a false discovery rate (FDR) q-value < 0.25 were considered statistically significant in this study.

### 2.5 Weighted gene co-expression network analysis

Weighted gene co-expression network analysis (WGCNA), a systems biology approach (29), was applied to analyze gene expression data from the GSE13355 and GSE75214 datasets. First, the top 25% of genes with the highest variance were selected to construct the input matrix, reducing noise and enhancing network robustness. Outlier samples and low-quality genes were filtered out using the “goodSamplesGenes” function from the WGCNA package to ensure data quality. Topological analysis was performed using the “pickSoftThreshold” function to determine the optimal soft-threshold power β, ranging from 1 to 20, which transformed the similarity matrix into a weighted adjacency matrix. A topological overlap matrix (TOM) was constructed, and gene clustering was performed using average linkage hierarchical clustering. Co-expressed gene modules were identified using the dynamic tree cut algorithm. The module eigengene (ME), representing the overall expression pattern of each module, was calculated. Pearson correlation analysis was then used to evaluate the association between the merged modules and disease occurrence, with statistical significance assessed using Student’s t-test. Modules showing the strongest positive and negative correlations with the disease were selected as the core modules for further analysis.

### 2.6 Functional enrichment analysis

Gene Ontology (GO) and Kyoto Encyclopedia of Genes and Genomes (KEGG) enrichment analyses were performed using the “ClusterProfiler” package (version 4.8.2) in R (30). GO is used to annotate biological processes, molecular functions, and cellular components, while KEGG is utilized for annotating gene pathways. Enrichment was considered statistically significant when *P* < 0.05.

### 2.7 Protein-protein interaction network construction and module analysis

Protein-protein interaction (PPI) analysis of the shared key genes was performed using the online tool STRING (https://string-db.org) (31). The filtering criteria were set to “highest confidence” with a confidence score threshold of >0.9, and isolated nodes were excluded from the network visualization. PPI networks were visualized using Cytoscape (v3.9.1) (32). The key gene clusters were screened by MCODE (Molecular Complex Detection), and the screening conditions were set to degree cutoff=2, node score cutoff=0.2, k-core=2, max depth=100.

### 2.8 Machine learning

To further identify shared hub genes, we employed six machine learning models, including Random Forest (RF), k-Nearest Neighbors (KNN), eXtreme Gradient Boosting (XGBoost), Decision Tree (Dtree), Support Vector Machine (SVM), and Least Absolute Shrinkage and Selection Operator (LASSO). Data preprocessing and model training were conducted in R using the “tidymodels” package (v1.2.0). The preprocessing steps included factorizing categorical variables, handling missing data with the “step_naomit()” function, and applying “step_dummy()” for one-hot encoding of categorical predictors. Model training was performed using a 5-fold cross-validation strategy to fine-tune hyperparameters, including the number of predictors (“mtry”) and the minimum node size (“min_n”). A grid search was conducted over a predefined hyperparameter space, and model performance was evaluated using three metrics: accuracy, ROC-AUC, and PR-AUC, computed with the “yardstick” package (v1.3.1). Although multiple evaluation metrics were considered, ROC-AUC was ultimately chosen as the primary criterion for hyperparameter selection. As a threshold-independent metric, ROC-AUC demonstrated the highest average value during cross-validation, providing a robust and consistent measure of model performance across varying classification thresholds (33). The final model trained with the optimal hyperparameter combination was then re-fitted on the entire training dataset and ROC curves for both the training and testing sets were generated to further assess model performance. Feature importance was analyzed using the “varImpPlot()” function, and partial dependence plots were generated to visualize the effects of key variables on classification outcomes.

### 2.9 Construction of receiver-operating characteristic curves to assess diagnostic efficacy

The “ROCR” package was utilized to generate the receiver operating characteristic (ROC) curve (34), evaluating the ability of shared hub genes to distinguish between psoriasis patients, CD patients, and healthy individuals across all datasets.

### 2.10 Immune infiltration analysis

Single-sample gene set enrichment analysis (ssGSEA) was performed using the GSVA package in R to evaluate the relative abundance of 28 immune cell types in psoriasis and CD samples. Pearson correlation analysis was performed to calculate the correlation between hub gene expression levels and immune cell abundance. The correlation coefficients were computed using the “rcorr()” function. Finally, a heatmap was generated to visualize the correlation between gene expression and immune cell abundance.

### 2.11 Identification of drug candidates

The shared hub genes of psoriasis and CD were input into the Enrichr platform (https://maayanlab.cloud/Enrichr/) (35). We then utilized the Drug Signature Database (DSigDB) to identify candidate drugs associated with the hub genes (36).

### 2.12 Molecular docking of candidate targets and active ingredients

We used the PubChem database (https://pubchem.ncbi.nlm.nih.gov/) to retrieve the chemical structures of compounds for docking against key proteins (37), including Etoposide, Lucanthone and Piroxicam. The crystal structure of KIF4A was obtained from the AlphaFold Protein Structure Database (38, 39), and the crystal structure of Cyclin B1 (CCNB1) was retrieved from the RCSB Protein Data Bank (PDB) (https://www.rcsb.org) (40). Molecular docking analyses were performed using the CB-Dock2 platform, which automatically predicted potential binding pockets and calculated the binding energies of the docking complexes (41, 42). The platform utilized an automatic scoring function to estimate binding affinities and ranked the docking conformations based on predicted binding scores. The results, including binding energies and docking poses, were further analyzed to evaluate the interaction patterns and affinities between the compounds and target proteins.

### 2.13 Cell culture

Human epidermal keratinocytes (HaCaT) were purchased from the Kunming Institute of Zoology, Chinese Academy of Sciences (Kunming, China), and the human colorectal adenocarcinoma cell line (HT-29) was obtained from the Chinese Academy of Sciences (Shanghai, China). Both cell types were cultured in Dulbecco’s Modified Eagle Medium (DMEM, Gibco, USA) supplemented with 10% fetal bovine serum (FBS, Gibco, USA) and 1% penicillin/streptomycin (Gibco, USA). Cells were incubated at 37°C in a 5% CO2 incubator and passaged when they reached approximately 80% confluence.

### 2.14 Establishment of psoriasis and CD cell models

HaCaT cells were treated with M5 (TNF-α, IL-17A, IL-22, IL-1α, and oncostatin M) at a concentration of 10 ng/mL for 24 hours to induce a psoriasis dermatitis inflammatory cell model (43). M5 cytokines sourced from PeproTech (Rocky Hill, USA) were utilized in this study. In addition, HT-29 cells were treated with 20 μg/mL lipopolysaccharide (LPS, Sigma-Aldrich, USA) for 24 hours to establish a CD inflammatory model (44).

### 2.15 Acquisition and preparation of Etoposide

Etoposide and dimethyl sulfoxide (DMSO) were both purchased from MedChemExpress (MCE, USA). Etoposide was initially dissolved in DMSO to prepare a 50 mM stock solution, which was aliquoted to avoid repeated freeze-thaw cycles and stored at -20°C. For *in vitro* experiments with HaCaT and HT-29 cell lines, the stock solution was freshly diluted with appropriate cell culture media to the desired working concentrations (e.g., 1, 5, 10, 20, and 50 μM) before use. All working solutions were freshly prepared, thoroughly mixed, and immediately applied to the cells to ensure drug stability and reproducibility of experimental results.

### 2.16 CCK-8 assay

The Cell Counting Kit-8 (CCK-8) was procured from Dojindo Molecular Technologies, Inc. (Kumamoto, Japan). HaCaT keratinocytes were cultured in a 96-well plate and subjected to treatment with M5 cocktail cytokines for durations ranging from 24 to 48 hours. Following treatment, each well received 10 μl of the CCK-8 reagent and the plate was incubated at 37°C for a period of 2 hours. The absorbance at 450 nm was subsequently quantified using a Multiskan microplate reader (Thermo Fisher Scientific).

### 2.17 Total RNA Extraction and Quantitative Real-Time PCR

HaCaT and HT-29 cells in the logarithmic growth phase were seeded in 6-well plates and stimulated as described. Cells were harvested 48 hours post-stimulation, and total RNA was extracted from approximately 1×10^6^ cells per well using TRIzol™ reagent (Invitrogen, USA). The extracted RNA was reverse-transcribed into complementary DNA (cDNA) using the RevertAid First Strand cDNA Synthesis Kit (Thermo Fisher Scientific, USA).

Quantitative real-time PCR (qRT-PCR) was performed using SYBR Green PCR Master Mix (TaKaRa, Japan) on a CFX96 Touch™ Real-Time PCR Detection System (Bio-Rad, USA). The thermocycling conditions were as follows: initial denaturation at 95°C for 10 s, annealing at 60°C for 10 s, and extension at 72°C for 10 s, for a total of 40 cycles. β-Actin (ACTB) served as an internal control, and relative gene expression levels were calculated using the 2^−ΔΔCt^ method. Specific primer sequences are provided in **Supplementary Table S1**.

### 2.18 Statistical analysis

The error bars in the figures represent the standard error of mean (SEM). For comparisons between two groups involving continuous variables, Student’s t-test was performed for normally distributed data. A one-way analysis of variance (ANOVA) was employed for multigroup comparisons. All statistical p-values were two-sided, with *P* < 0.05 considered statistically significant.

## 3 Result

### 3.1 Identification of differentially expressed genes in psoriasis and CD

**Figure 2:**
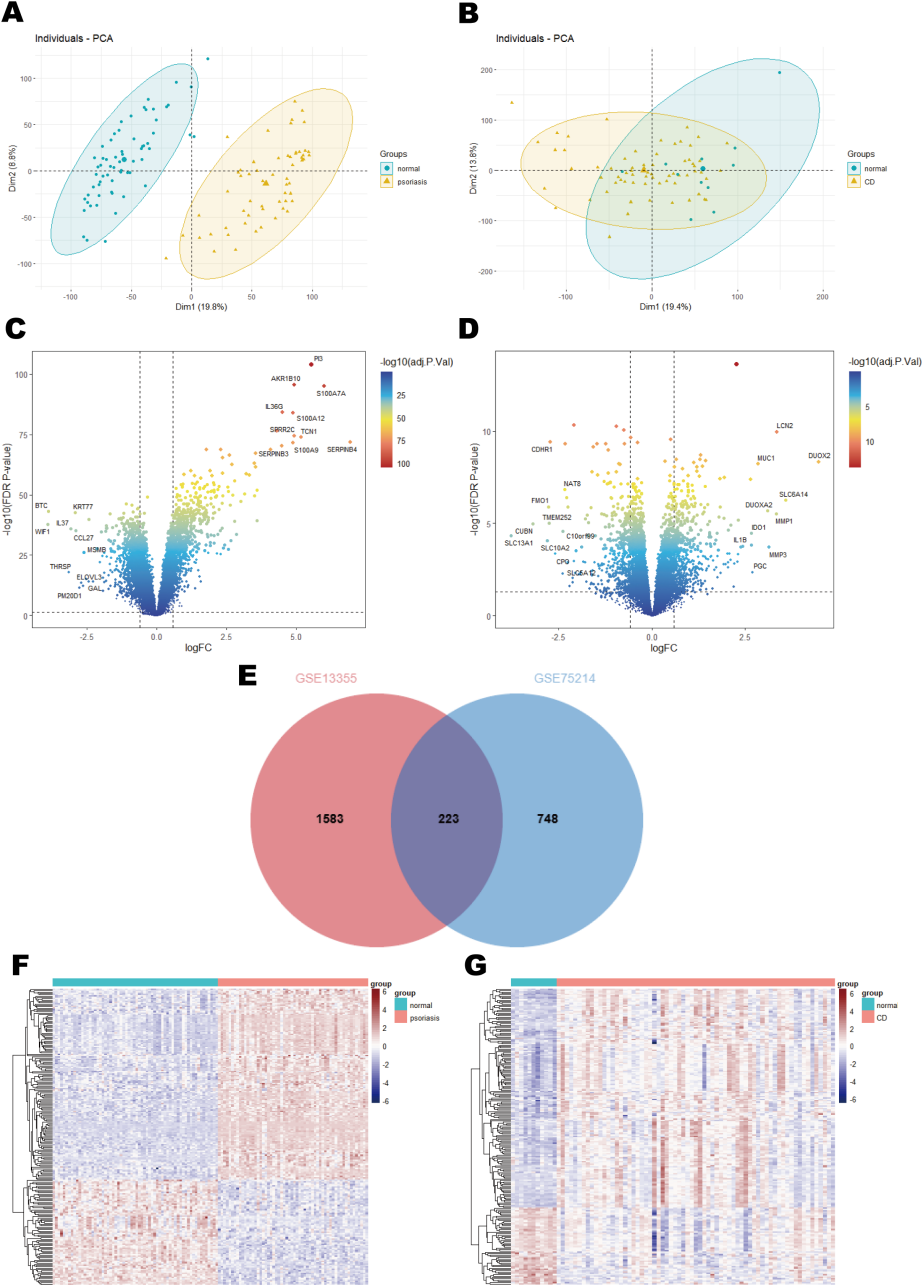
Results of differential expression analysis of psoriasis and CD. **(A)** Principal component analysis in GSE13355. **(B)** Principal component analysis in GSE75214. **(C)** A volcano plot of DEGs in GSE13355. **(D)** A volcano plot of DEGs in GSE75214. **(E)** Venn diagram of shared DEGs in psoriasis and CD. **(F)** A heatmap of the shared DEGs in GSE13355. **(G)** A heatmap of the shared DEGs in GSE75214.

Principal component analysis (PCA) was applied to assess sample variations in the psoriasis (GSE13355) and CD (GSE75214) datasets, revealing distinct separation between patient groups and healthy controls for both conditions (**Figures 2A, B**). From the psoriasis dataset, differential expression analysis revealed 1,806 differentially expressed genes (DEGs), including 812 upregulated and 994 downregulated genes (**Figure 2C**). Similarly, 971 DEGs were detected in the CD dataset, comprising 557 upregulated and 414 downregulated genes (**Figure 2D**). Importantly, 223 overlapping DEGs were shared between the two datasets (**Figure 2E**), with their expression profiles visualized in a heatmap. Specifically, **Figure 2F** illustrates the distinct expression patterns of these DEGs in 58 psoriasis patients compared to 64 healthy controls, while **Figure 2G** highlights their differential expression in 67 CD samples versus 11 control samples.

### 3.2 Gene set enrichment analysis

**Figure 3:**
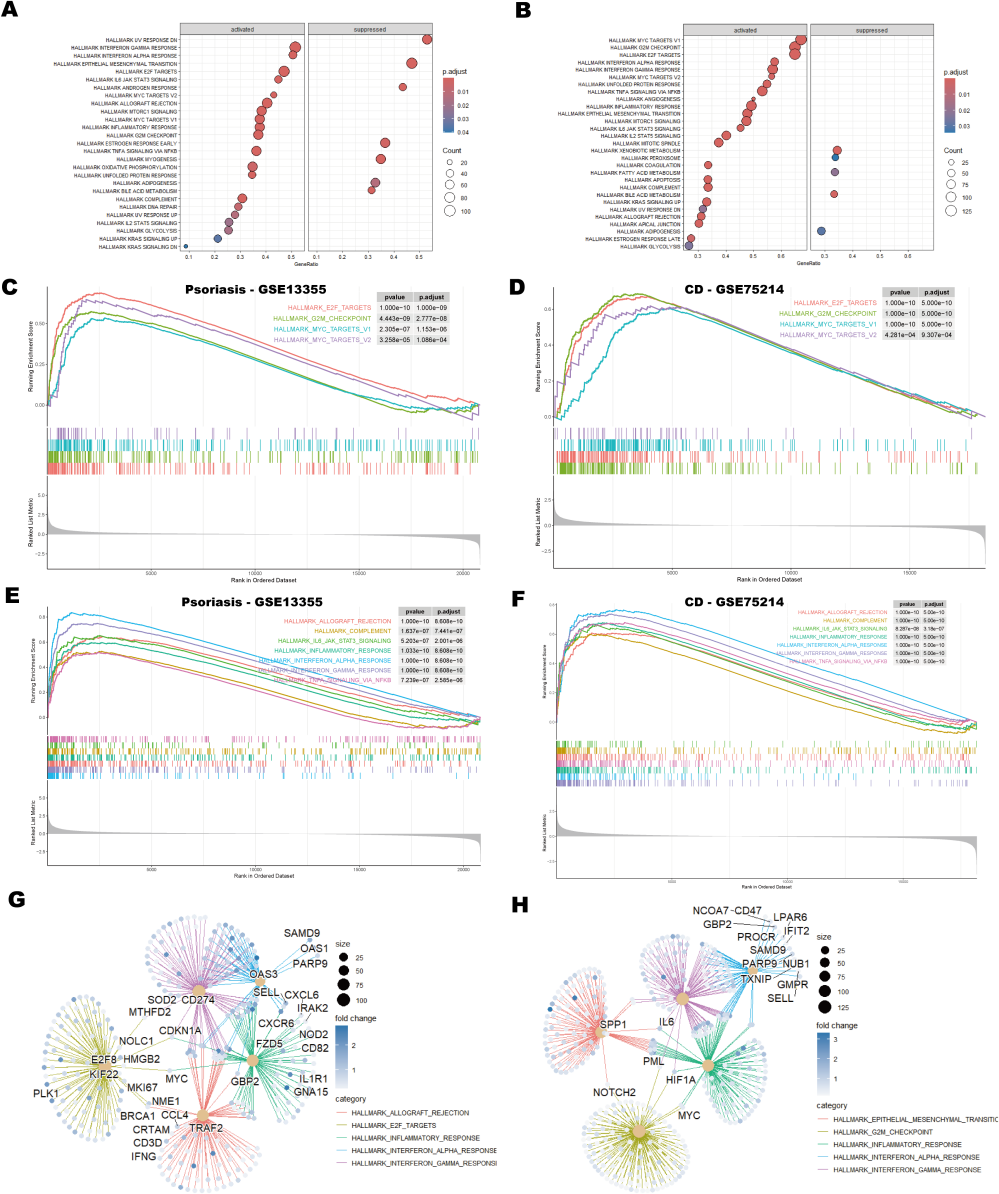
GSEA analysis of psoriasis and CD datasets. **(A, B)** Bubble plots showing GSEA enrichment results for psoriasis (GSE13355) and CD (GSE75214). The size of the bubbles represents the number of genes (counts) in each enriched pathway, and the color intensity indicates the enrichment significance, as measured by the adjusted p-values. **(C, D)** GSEA enrichment plots depicting cell cycle-related pathways in psoriasis and CD, showing the enrichment score for each gene set along with the corresponding p-values. **(E, F)** GSEA enrichment plots illustrating immune-related pathways in psoriasis and CD. These plots provide insight into the immune response-related gene sets enriched in the datasets. **(G, H)** Protein-protein interaction (PPI) network analysis of genes associated with the top five pathways in psoriasis and CD. The network analysis identifies key hub genes involved in the top-ranked pathways, showing their interactions and potential roles in disease progression. Hub genes are indicated in larger nodes, and edges represent significant protein-protein interactions.

Gene Set Enrichment Analysis (GSEA) using the HALLMARK gene sets identified significant downregulation of the HALLMARK_UV_RESPONSE_DN and HALLMARK_EPITHELIAL_MESENCHYMAL_TRANSITION (EMT) pathways in the psoriasis dataset (**Figures 3A, B**). Suppression of the UV_RESPONSE_DN pathway suggests impaired cellular responses to UV-induced stress, which may contribute to immune dysregulation, deficiencies in DNA repair, and abnormal cell proliferation, collectively exacerbating the pathogenesis of psoriasis (45). Similarly, downregulation of the EMT pathway may impair skin repair and regeneration, restrict keratinocytes migration, and delay wound healing. This inhibition could contribute to skin barrier dysfunction and increased immune cell infiltration, thereby exacerbating epidermal thickening and inflammation (46, 47).

In both the psoriasis and CD datasets, pathways associated with the cell cycle, including HALLMARK_E2F_TARGETS, HALLMARK_G2M_CHECKPOINT, HALLMARK_MYC_TARGETS_V1, and HALLMARK_MYC_TARGETS_V2, showed significant enrichment (**Figures 3C, D**). This finding suggests that dysregulation of the cell cycle is a shared pathological mechanism in both diseases, particularly in the context of immune-mediated chronic inflammation. Aberrations in E2F and G2/M checkpoints may drive excessive proliferation of keratinocytes in psoriasis and intestinal epithelial cells in CD, while activation of the MYC pathway may amplify pathological cell proliferation (48–51).

Moreover, inflammation-related pathways were prominently enriched in both diseases. The activation of HALLMARK_INTERFERON_GAMMA_RESPONSE and HALLMARK_INTERFERON_ALPHA_RESPONSE pathways underscores the critical role of interferon signaling in modulating immune responses (52) (**Figures 3E, F**). Additionally, pathways such as HALLMARK_INFLAMMATORY_RESPONSE and HALLMARK_TNFA_SIGNALING_VIA_NFKB directly contribute to the maintenance and amplification of inflammation. Notably, the enrichment of HALLMARK_IL6_JAK_STAT3_SIGNALING highlights the potential role of the IL-6/STAT3 axis in linking immune cell activation with abnormal cell proliferation, which may exacerbate disease progression (53) (**Figures 3E, F**). **Figures 3G, H** illustrate the five most enriched pathways alongside the corresponding protein-protein interaction (PPI) networks identified in psoriasis and CD, respectively. In **Figure 3G**, pathways such as allograft rejection, E2F targets, inflammatory response, and interferon responses are associated with hub genes TRAF2, GBP2 and CD274, which are crucial for immune regulation. **Figure 3H** highlights pathways including EMT, G2/M checkpoint, inflammatory response and interferon responses, with core genes SPP1, IL6, MYC, and HIF1A governing critical regulatory mechanisms. In summary, the co-enrichment of these cell cycle and inflammation-related pathways underscores the shared dysregulation of immune responses and cell proliferation in psoriasis and CD.

### 3.3 WGCNA identifies key modules in psoriasis and CD

**Figure 4:**
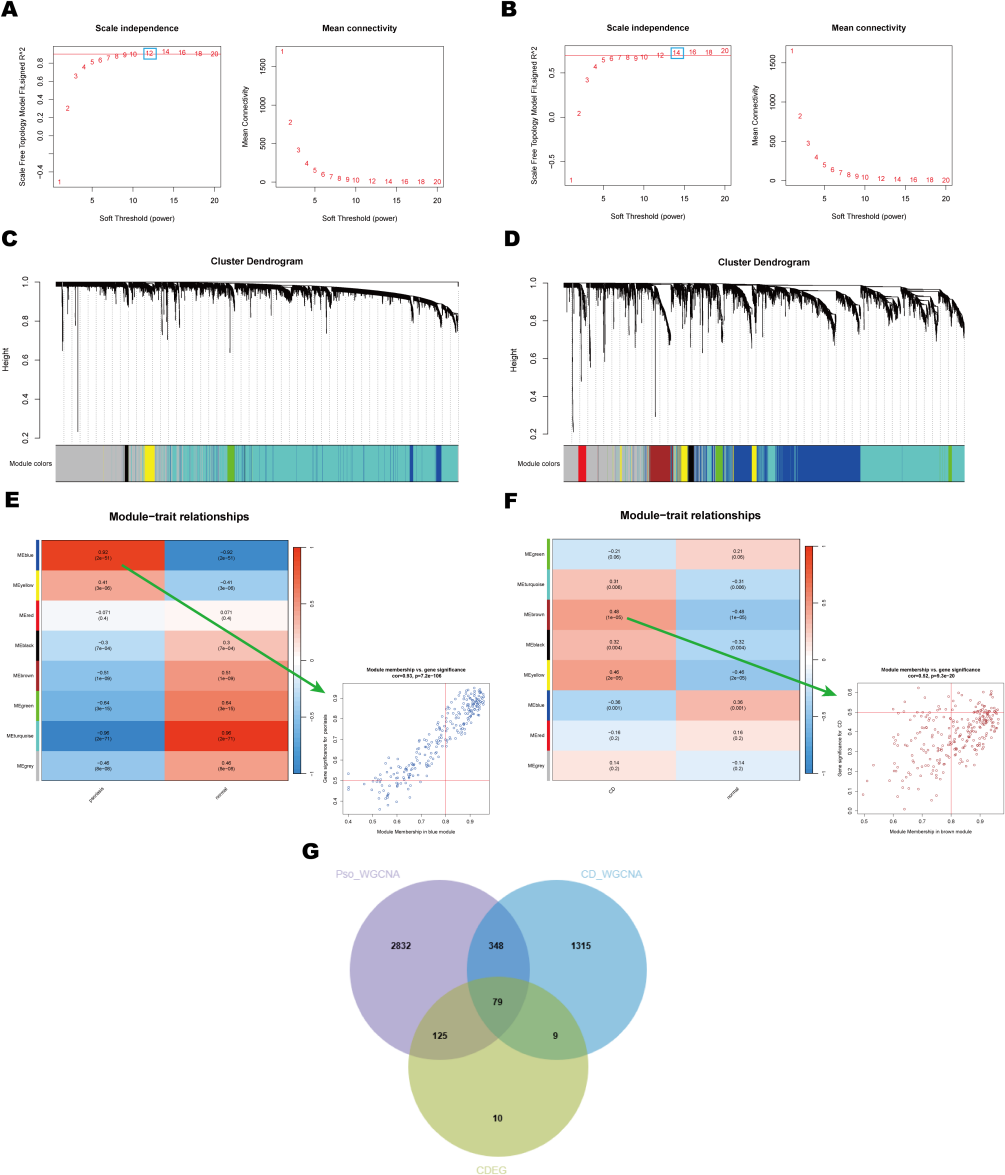
Identification and analysis of key module of psoriasis and CD by WGCNA. **(A, B)** Scale independence and average connectivity plots for psoriasis and CD. These plots illustrate the selection of the soft threshold power (β) for network construction in WGCNA. **(C, D)** Clustering dendrograms of module feature genes in psoriasis and CD. These dendrograms show the hierarchical clustering of genes based on their module feature values, identifying distinct gene modules that are strongly associated with disease phenotypes in both psoriasis and CD. **(E, F)** Heatmaps of module-trait correlations and scatter plots for the modules with the highest correlation in psoriasis and CD. Each row represents a color module, and every column represents a clinical trait. The correlation coefficient and corresponding P-value are shown in each cell. **(G)** Venn diagram showing shared DEGs and genes in related modules in psoriasis and CD. This diagram highlights the overlap of DEGs across both diseases, identifying key genes that may play a role in the pathogenesis of both psoriasis and CD.

To investigate potential associations between diseases and genes, WGCNA was conducted on the psoriasis dataset (GSE13355) and the CD dataset (GSE75214). Using the WGCNA framework, the optimal soft-thresholding power was determined to be 12 for the psoriasis dataset and 14 for the CD dataset (**Figures 4A, B**). Module similarity analysis identified eight significant modules in both datasets (**Figures 4C, D**). Heatmap analysis of module-trait relationships revealed that the blue module showed the strongest positive correlation with psoriasis (r = 0.92), while the turquoise module exhibited the most pronounced negative correlation (r = -0.96) (**Figure 4E**). In the CD dataset, the brown module showed the strongest positive correlation with CD (r = 0.48), and the blue module demonstrated the strongest negative correlation (r = -0.36) (**Figure 4F**).

Notably, there was a significant correlation between Gene Significance (GS) and Module Membership (MM) within modules, with correlation coefficients of 0.92 in the psoriasis dataset and 0.48 in the CD dataset (**Figures 4E, F**). This finding indicates a robust association between the identified module genes and disease pathogenesis. By intersecting 223 common DEGs (CDEG) with the 427 genes derived from WGCNA, a total of 79 shared key genes were identified as candidates for further analysis (**Figure 4G**). These shared key genes are hypothesized to play pivotal roles in the pathogenesis and progression of both psoriasis and CD.

### 3.4 GO and KEGG enrichment analyses were conducted to identify biological processes and signaling pathways associated with shared key genes

**Figure 5:**
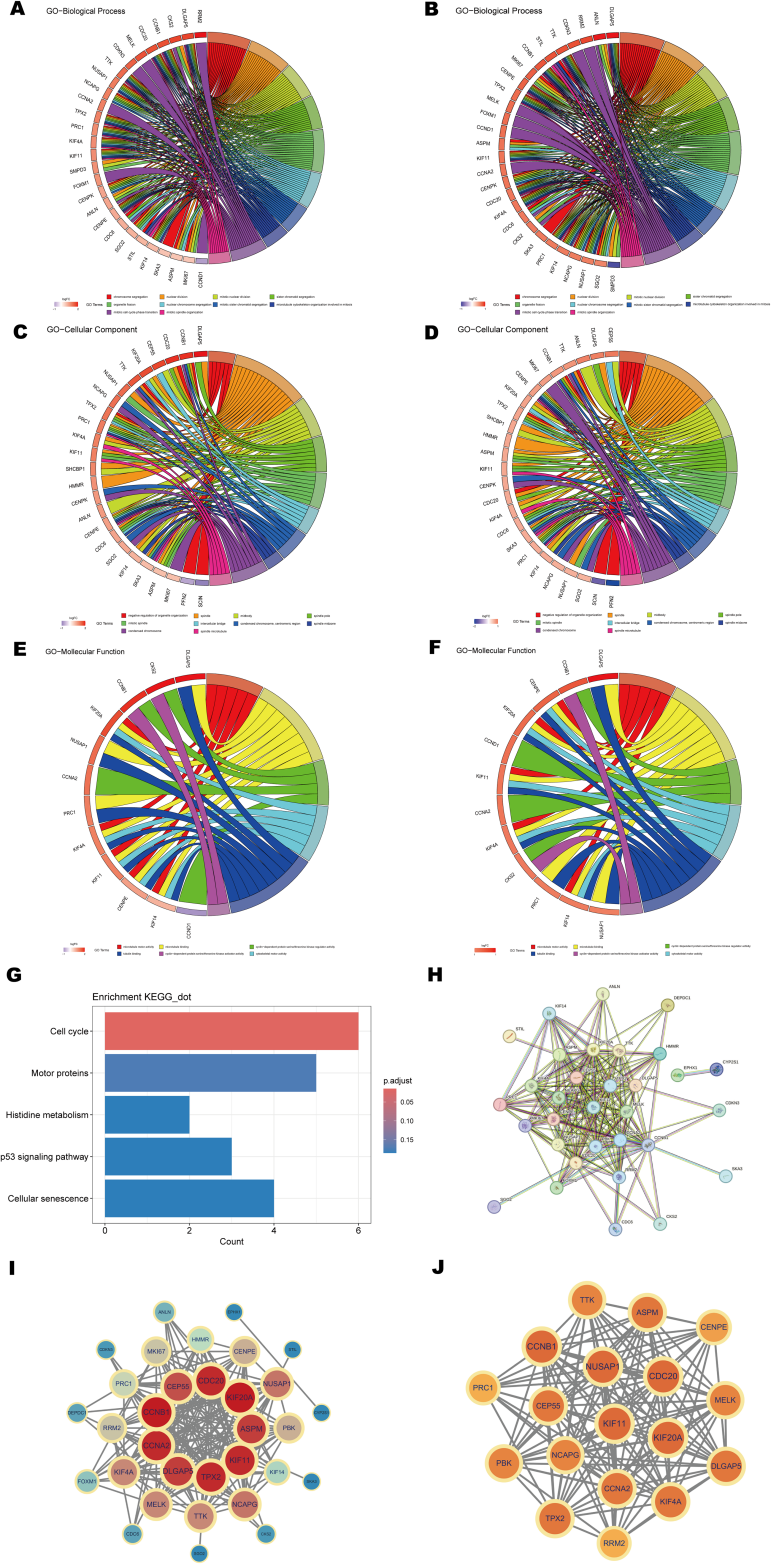
Functional enrichment analysis and PPI network construction. **(A, B)** Chord diagrams of shared key genes enriched in the GO Biological Process (BP) category for psoriasis and CD. **(C, D)** Chord diagrams of shared key genes enriched in the GO Cellular Component (CC) category for psoriasis and CD. **(E, F)** Chord diagrams of shared key genes enriched in the GO Molecular Function (MF) category for psoriasis and CD. **(G)** Bar plot of KEGG pathway enrichment analysis. **(H)** PPI network of shared key genes constructed using the STRING database. **(I, J)** Key modules and hub genes identified in the PPI networks using the MCODE algorithm.

To further elucidate the biological functions of the shared key genes, GO and KEGG enrichment analyses were conducted. The GO analysis results, visualized using chord diagrams (**Figures 5A–F**), emphasized Biological Processes (BP) such as chromosome segregation (GO:0007059), nuclear division (GO:0000280), mitotic nuclear division (GO:0140014), and sister chromatid separation (GO:0000819) (**Figures 5A, B**). These processes were significantly enriched, with adjusted p-values < 0.05, highlighting the essential roles of these genes in cell cycle regulation, mitosis, and chromosome allocation. KEGG pathway enrichment analysis further revealed significant enrichment in the cell cycle pathway (**Figure 5G**), suggesting that dysregulation of cell division and cell cycle checkpoint control may represent a shared pathogenic mechanism in psoriasis and CD. In summary, GO and KEGG analyses underscore the pivotal roles of shared key genes in cell cycle-related processes and indicate their likely contribution to the shared pathological mechanisms underlying these diseases. These findings provide valuable insights into the molecular basis of co-pathogenesis, particularly through the regulation of chromosome segregation and mitosis.

### 3.5 Key genes of the PPI network and identification of hub genes

To investigate the interactions between proteins encoded by the shared key genes, a protein-protein interaction (PPI) network was generated using the STRING database with a confidence score threshold of >0.9 (**Figure 5H**). The network, visualized using Cytoscape (v3.9.1), comprised 32 nodes and 204 edges (**Figure 5I**). Modular analysis, performed using the MCODE plugin, identified a core module containing 18 hub genes with a cluster score of 16.353 (**Figure 5J**). The hub genes include PRC1, NUSAP1, CCNA2, PBK, DLGAP5, KIF4A, KIF11, TTK, ASPM, TPX2, CDC20, CEP55, KIF20A, NCAPG, CCNB1, CENPE, RRM2, and MELK. These genes are critical regulators of fundamental biological processes, including cell cycle progression, mitosis, and inflammation, all of which are integral to the pathogenesis of both psoriasis and CD. Specifically, CCNA2, CDC20, and CCNB1 are involved in regulating the G2/M phase transition and mitotic progression (54), suggesting their potential contribution to the abnormal cell proliferation observed in both diseases. Additionally, KIF4A, KIF11, and KIF20A are linked to mitotic spindle formation and chromosome segregation, processes often disrupted in hyperproliferative conditions such as psoriasis and CD. Furthermore, genes such as PBK and CEP55 are vital in regulating inflammation and immune responses, potentially influencing immune cell dynamics and the inflammatory microenvironment in these diseases. TPX2 and RRM2 are involved in DNA damage repair and stress signaling, processes essential for maintaining cellular integrity in the context of inflammation and tissue damage.

The identification of this core module and its hub genes highlights their centrality and regulatory significance in the shared molecular mechanisms underlying both diseases. These findings suggest that these hub genes may represent potential therapeutic targets for managing psoriasis and CD.

### 3.6 Identification and validation of potential shared biomarkers through multiple machine learning approaches

**Figure 6:**
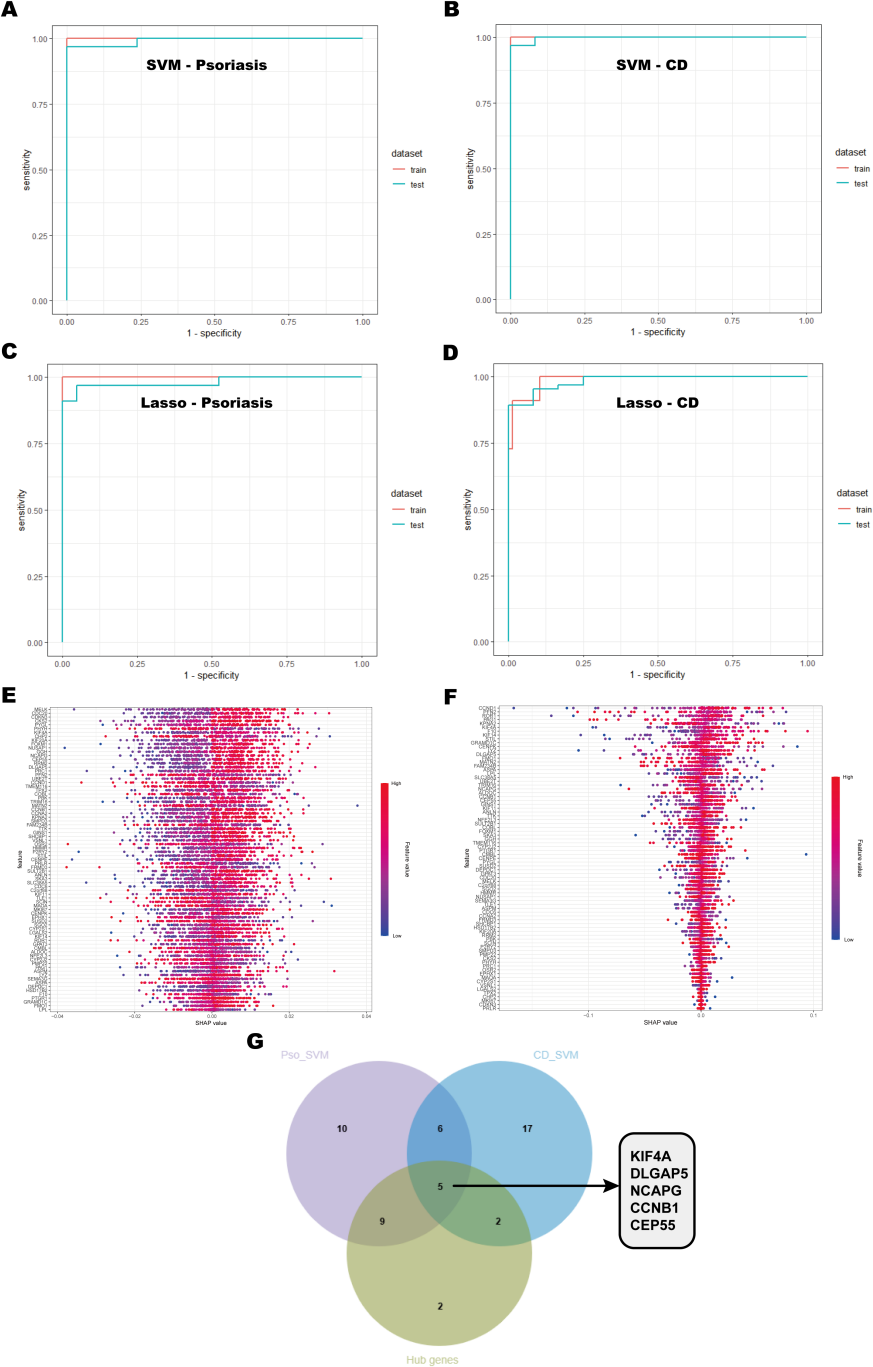
Machine learning model selection and hub gene identification. **(A, B)** ROC curves of the SVM model for psoriasis and CD, showing performance on the training and testing sets. The area under the curve (AUC) values are provided to assess the model’s discriminative ability. **(C, D)** ROC curves for the Lasso model applied to psoriasis and CD. **(E, F)** SHAP value plots highlighting the importance of gene features in the SVM model. Each point represents the impact of a gene’s expression on the model’s output, with the x-axis showing the SHAP value and the y-axis showing the gene features. **(G)** Venn diagram showing the overlap of key genes identified by SVM and hub genes from the PPI network.

To further identify the most diagnostically valuable shared biomarkers, six machine learning algorithms, including RF, KNN, XGBoost, Dtree, SVM, and Lasso, were employed to select feature genes from the 79 shared key genes identified in earlier analyses. The performance of each model was systematically evaluated by generating ROC curves for both the training and test datasets (**Supplementary Figure S1**). Among these machine learning models, the SVM model achieved the highest predictive performance in both psoriasis and CD datasets (**Figures 6A, B**), while the Lasso model also exhibited robust diagnostic accuracy (**Figures 6C, D**). To improve the interpretability of the SVM model, Shapley Additive Explanations (SHAP) values were employed to assess the importance of individual features (**Figures 6E, F**). A feature value-SHAP value correlation plot was generated to illustrate each feature’s contribution to the model’s output. Based on the SHAP analysis, the top 30 genes were selected based on their importance in the SVM model and intersected with the 18 hub genes identified from the PPI network using the MCODE algorithm (**Figure 6G**). This integrative approach highlighted KIF4A, DLGAP5, NCAPG, CCNB1, and CEP55 as key shared biomarkers for both psoriasis and CD.

### 3.7 Validation of shared hub genes with GEO databases

**Figure 7:**
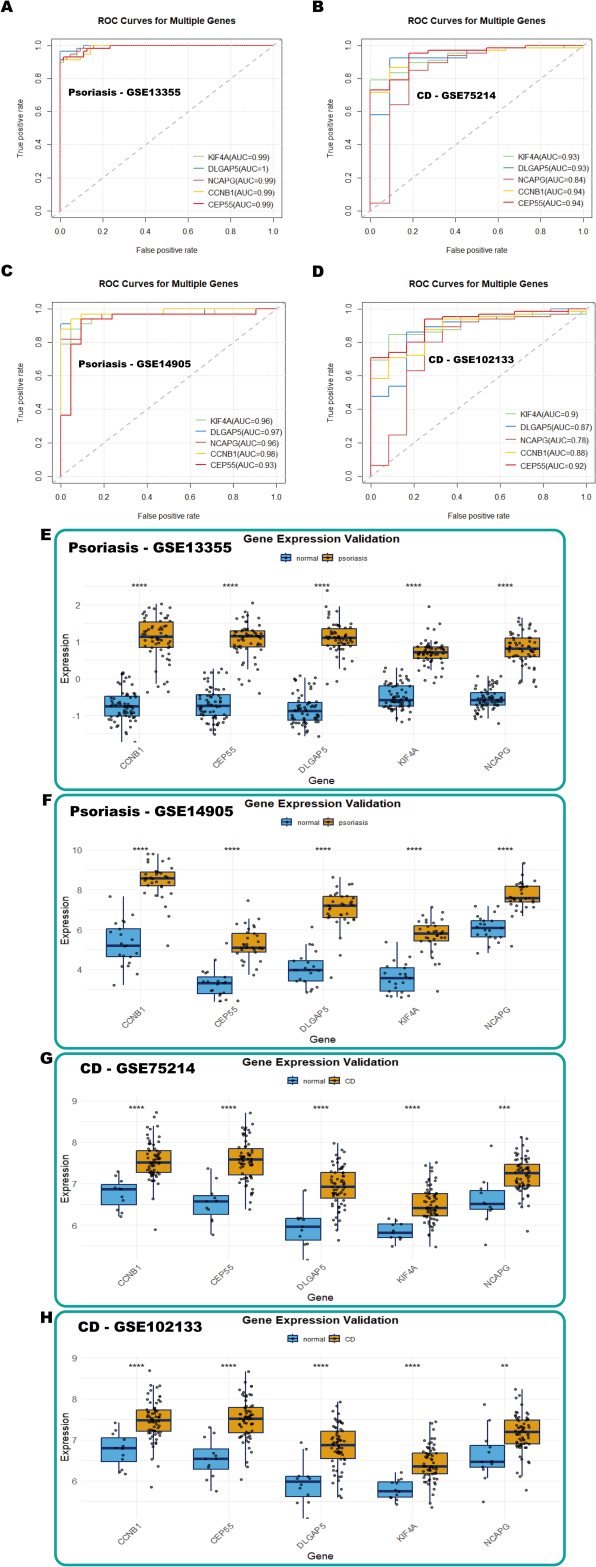
Validation of hub genes with GEO databases. **(A-D)** ROC curves of hub genes in GSE13355 (psoriasis), GSE75214 (CD), GSE14905 (psoriasis), and GSE102133 (CD), respectively. **(E-H)** Box plots showing hub gene expression in GSE13355 (psoriasis), GSE14905 (psoriasis), GSE75214 (CD), and GSE102133 (CD), respectively.

The diagnostic predictive value of the identified hub genes was evaluated using ROC curve analysis across multiple datasets. In the psoriasis dataset (GSE13355), the AUC values for KIF4A (AUC = 0.99), DLGAP5 (AUC = 1), NCAPG (AUC = 0.99), CCNB1 (AUC = 0.99), and CEP55 (AUC = 0.99) exhibited exceptional diagnostic performance, with all values exceeding 0.7 (**Figure 7A**). Similarly, in the CD dataset (GSE75214), the AUC values for KIF4A (AUC = 0.93), DLGAP5 (AUC = 0.93), NCAPG (AUC = 0.84), CCNB1 (AUC = 0.94), and CEP55 (AUC = 0.94) also exceeded 0.7, further supporting their robust diagnostic potential (**Figure 7B**). To validate these findings, the predictive efficacy of these biomarkers was examined in independent validation cohorts. In the psoriasis validation cohort (GSE14905), the AUC values for KIF4A, DLGAP5, NCAPG, CCNB1, and CEP55 were 0.96, 0.97, 0.96, 0.98, and 0.93, respectively. Likewise, in the CD validation cohort (GSE102133), the AUC values for these genes were 0.90, 0.87, 0.78, 0.88, and 0.92, respectively, with all markers exhibiting AUC values above 0.7, reaffirming their significance as diagnostic biomarkers (**Figures 7C, D**).

Furthermore, box plot analyses revealed significant upregulation of these five diagnostic markers in the disease groups compared to the controls in both psoriasis and CD training datasets (**Figures 7E, G**). Consistent differential expression patterns were observed in the psoriasis (GSE14905) and CD (GSE102133) validation cohorts (**Figures 7F, H**). These findings collectively underscore the potential of KIF4A, DLGAP5, NCAPG, CCNB1, and CEP55 as shared diagnostic biomarkers for psoriasis and CD.

### 3.8 Immune cell infiltration and its correlation with shared hub genes

**Figure 8:**
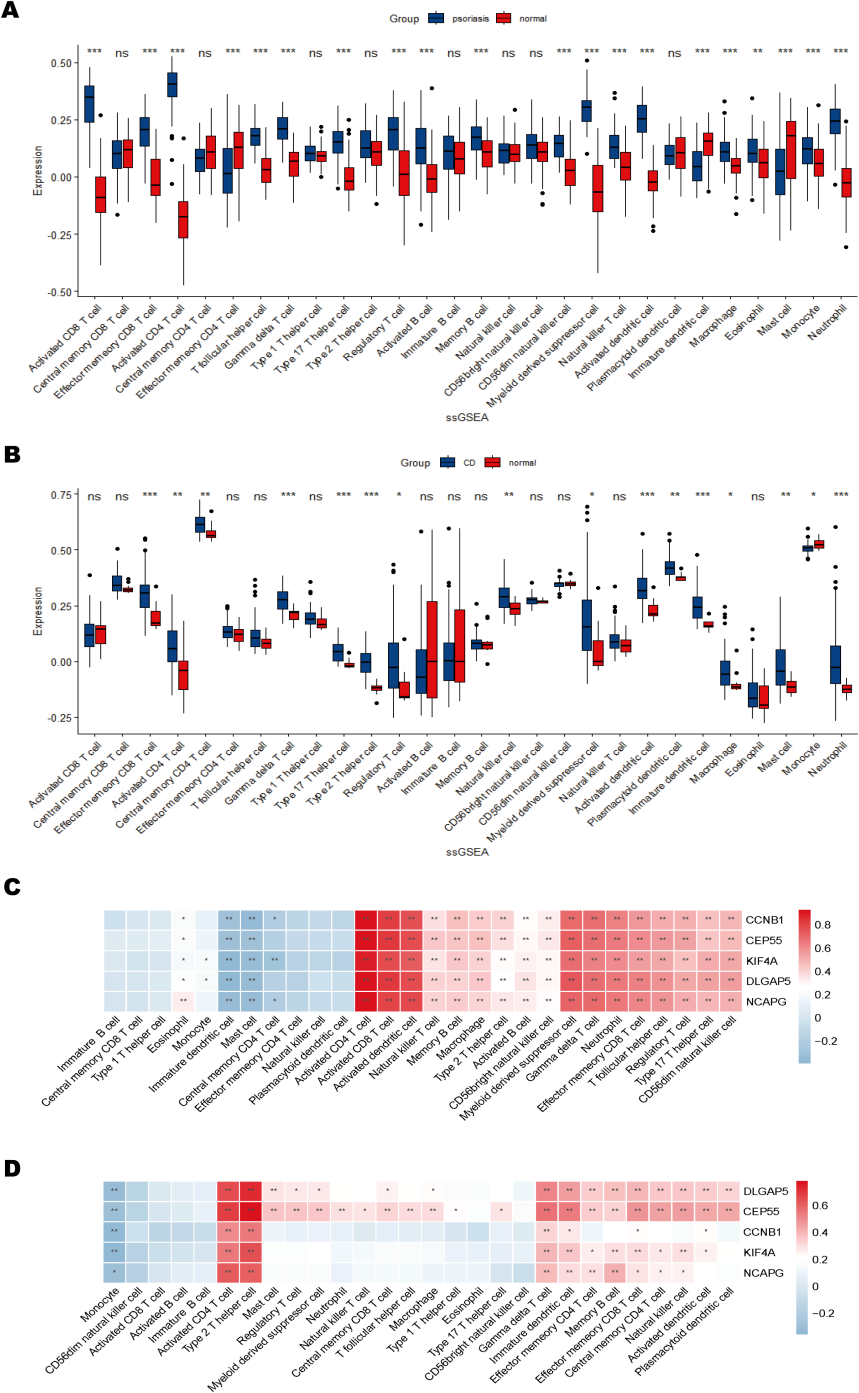
Immune cell infiltration and correlation with shared hub genes. **(A, B)** Box plots of immune cell infiltration differences in psoriasis and CD compared to normal controls. **(C, D)** Heatmaps of correlations between immune cells and shared hub genes in psoriasis and CD. The color scale indicates the strength of the correlation, with positive correlations in red and negative correlations in blue. *P < 0.05; **P < 0.01; ***P < 0.001.

Single-sample Gene Set Enrichment Analysis (ssGSEA) was used to evaluate immune cell infiltration in psoriasis and CD. The analysis revealed significantly elevated immune cell infiltration in psoriasis patients compared to normal controls, with 20 out of 28 immune cell types exhibiting elevated infiltration levels in psoriasis samples (**Figure 8A**). Similarly, in CD patients, immune cell infiltration was generally higher compared to controls, with 16 immune cell types showing significant increases (**Figure 8B**). Notably, 10 immune cell types demonstrated consistently higher infiltration levels in both psoriasis and CD samples relative to controls.

The correlations between immune cell infiltration and hub gene expression were further analyzed (**Figures 8C, D**). The results indicated significant positive correlations between most immune cell types and the hub genes. Specifically, in the psoriasis dataset, hub genes KIF4A, DLGAP5, NCAPG, CCNB1, and CEP55 were strongly positively correlated with activated CD4+ and CD8+ T cells, activated dendritic cells, myeloid-derived suppressor cells (MDSCs), and γδT cells, while negatively correlated with immature dendritic cells and mast cells (**Figure 8C**). In the CD dataset, these five hub genes were predominantly positively correlated with activated CD4+ T cells and Th2 cells, and negatively correlated with monocytes (**Figure 8D**). These findings suggest that the hub genes may contribute to autoimmune regulation by modulating the activation or suppression of specific immune cell populations. Collectively, the immune infiltration analysis highlights both commonalities and differences in immune cell infiltration between psoriasis and CD, emphasizing the potential role of hub genes in immune system regulation and their involvement in modulating autoimmune responses.

### 3.9 Single-cell analysis of hub gene locations

To investigate the cellular-level gene expression characteristics and alterations in psoriasis and CD, single-cell RNA sequencing (scRNA-seq) datasets (GSE162183 for psoriasis and GSE214695 for CD) were integrated and reanalyzed. After rigorous quality control, 15,592 cells were retained in the psoriasis dataset, and 23,591 cells were retained in the CD dataset (**Supplementary Figure S2**).

**Figure 9:**
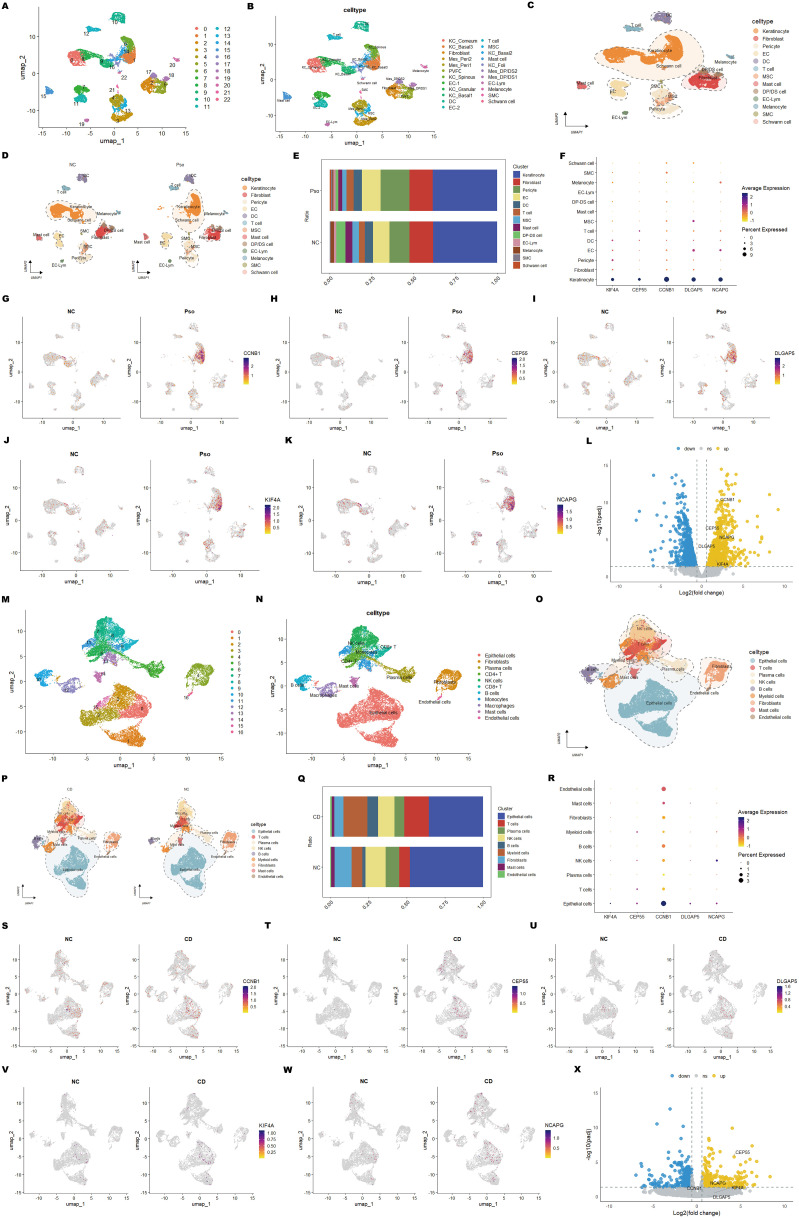
Single-cell RNA sequencing reveals cell-type-specific expression of core genes in psoriasis and CD. **(A)** UMAP plot showing cell clusters identified in psoriasis samples. **(B)** UMAP plot annotated with specific cell types in psoriasis samples. **(C)** Visualization of major cell types in psoriasis samples. **(D)** Comparison of cell type composition between psoriasis samples and normal controls. **(E)** Stacked bar plot displaying the distribution of cell types across psoriasis samples and normal controls. **(F)** Dot plot showing the average expression and the percentage of cells expressing shared hub genes across cell types. **(G-K)** UMAP feature plots illustrating the expression patterns of hub genes in psoriasis samples and normal controls. **(L)** Volcano plot showing the differential expression of hub genes between psoriasis samples and normal controls in keratinocytes. **(M-X)** Equivalent analyses performed on CD datasets, including cell cluster annotation, cell-type distribution, gene expression patterns, and differential expression analysis in epithelial cells.

In the psoriasis dataset (GSE162183), unsupervised clustering via UMAP identified 23 distinct cell clusters. Based on annotations from the original publication and related studies (55, 56), these clusters were further classified into 23 subtypes (**Supplementary Figure S3**), visualized on UMAP plots (**Figures 9A, B**). These subtypes were further consolidated into 13 major cell types: keratinocytes (5,986 cells), fibroblasts (2,188 cells), pericytes (2,252 cells), endothelial cells (1,625 cells), dendritic cells (758 cells), T cells (610 cells), mesenchymal stem cells (512 cells), mast cells (502 cells), dermal papilla/dermal sheath cells (584 cells), EC-lymphocytes (205 cells), melanocytes (204 cells), smooth muscle cells (102 cells), and Schwann cells (64 cells) (**Figure 9C**). UMAP plots illustrated the distribution of these cell types between psoriasis patients and healthy controls (**Figure 9D**).

Similarly, for the CD dataset (GSE214695), UMAP clustering revealed 17 cell clusters (**Figure 9M**). These clusters were annotated into 11 distinct cell types based on the original publication and additional references (50) (**Figure 9N**) (**Supplementary Figure S3**). These cell types were further grouped into 9 major categories: epithelial cells (9,846 cells), T cells (2,730 cells), plasma cells (1,770 cells), natural killer (NK) cells (2,832 cells), B cells (1,086 cells), myeloid cells (2,651 cells), fibroblasts (1,994 cells), mast cells (495 cells), and endothelial cells (187 cells) (**Figure 9O**). UMAP plots similarly mapped the distribution of these cell types between CD patients and healthy controls (**Figure 9P**).

Subsequently, cellular composition between patient and control groups was compared to identify key subpopulations implicated in the pathogenesis of psoriasis and CD. In psoriasis, keratinocytes abundance showed no significant difference, yet plasma cell numbers were markedly increased (**Figure 9E**). This suggests that while keratinocytes undergo hyperproliferation and abnormal differentiation, their overall abundance remains stable. Psoriatic lesions are characterized by hyperkeratosis and epidermal thickening (2), primarily due to enhanced keratinocyte proliferation. Immune cell infiltration, including T cells, dendritic cells, and plasma cells, plays a pivotal role in driving inflammation. The elevated plasma cell numbers likely reflect heightened B cell activation and plasma cell generation, accompanied by increased secretion of pro-inflammatory cytokines such as IL-17 and IL-22, which collectively exacerbate inflammation and contribute to psoriatic skin lesions.

In CD, epithelial, endothelial, and NK cell proportions were significantly reduced (**Figure 9Q**), indicating compromised barrier integrity, vascular dysfunction, and impaired immune surveillance under chronic inflammation. Conversely, T cell and myeloid cell proportions were significantly elevated (**Figure 9Q**), highlighting excessive immune cell recruitment and activation that perpetuate the inflammatory response. Reduced epithelial and endothelial cell abundance points to impaired tissue repair, while elevated T cell and myeloid cell numbers underscore the pro-inflammatory microenvironment. These findings reveal the complex interplay between immune dysregulation and tissue damage in CD.

To further understand the cellular context of the diagnostic markers identified in psoriasis and CD, their spatial distribution and expression patterns across different cell types were analyzed. In psoriasis, the five diagnostic hub genes (KIF4A, DLGAP5, NCAPG, CCNB1, and CEP55) were predominantly expressed in keratinocytes (**Figure 9F**), with significantly higher expression levels in patient samples compared to controls (**Figures 9G–K**). This observation highlights their critical roles in regulating keratinocyte proliferation and differentiation. Subsequently, the keratinocyte population was isolated for differential analysis, and the volcano plot visualized the differentially expressed genes between psoriasis samples and normal controls (**Figure 9L**). In CD, these hub genes were highly expressed in epithelial cells (**Figures 9R, S–W**), implicating their involvement in epithelial cell proliferation and barrier maintenance. Interestingly, NCAPG also exhibited high expression in NK cells, suggesting a potential role in intestinal immune responses and NK cell-mediated cytotoxicity. Similarly, the epithelial cell population was isolated for differential analysis, and the volcano plot visualized the differentially expressed genes between CD samples and normal controls, with CEP55 showing the most significant difference (**Figure 9X**).

The consistent upregulation of these five hub genes in both diseases indicates shared pathological mechanisms, including cell cycle dysregulation and aberrant immune responses. These findings highlight the potential of these genes as shared diagnostic markers and therapeutic targets for psoriasis and CD. Furthermore, their distinct cellular expression patterns underscore their contributions to tissue-specific pathophysiology. These results provide a solid foundation for the development of diagnostic panels and therapeutic strategies targeting the shared molecular mechanisms underlying psoriasis and CD.

### 3.10 Validation of shared hub genes via cellular experiments

**Figure 10:**
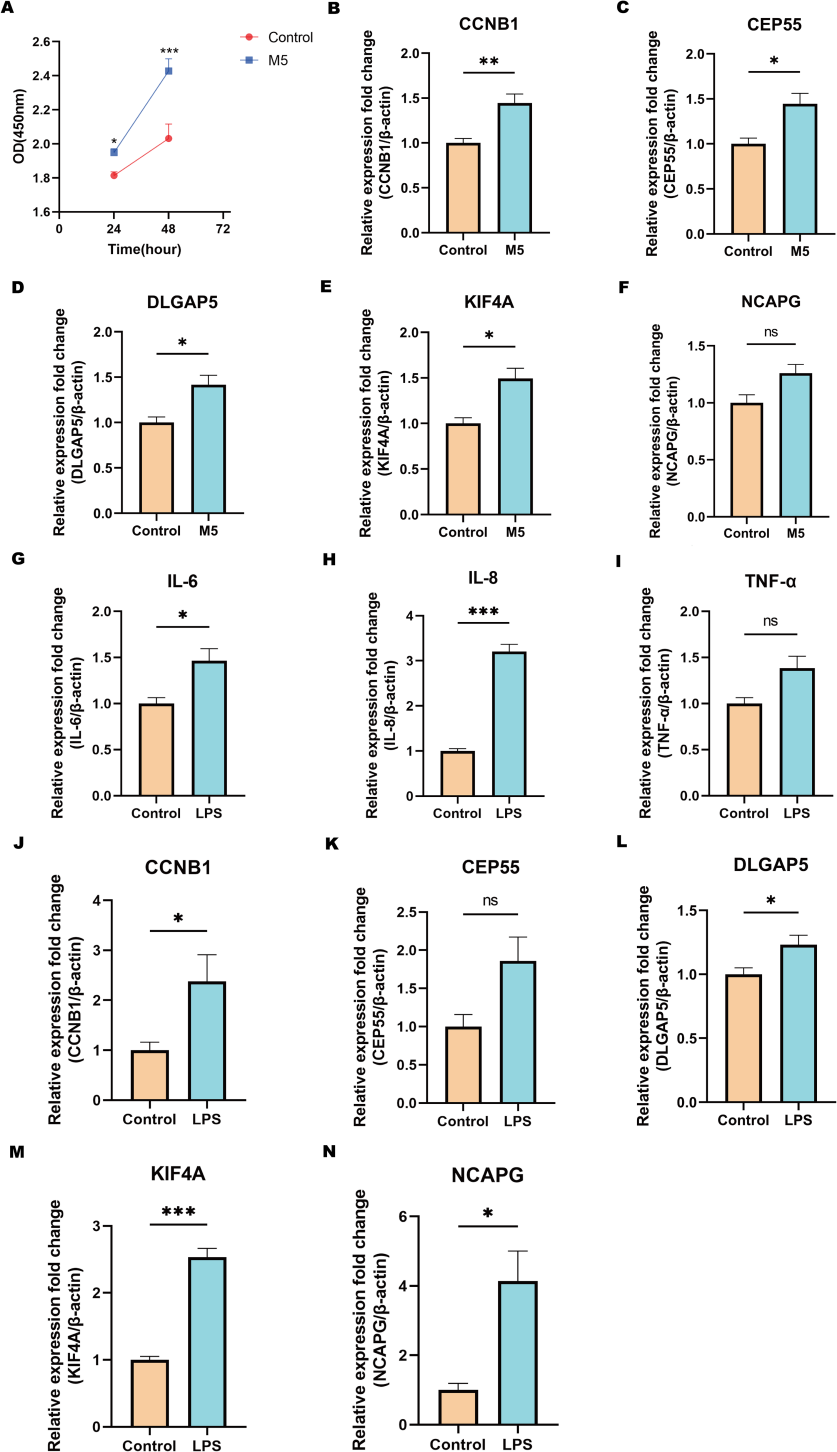
Validation of hub genes via cellular experiments. **(A)** CCK-8 assay showing the proliferation capacity (OD value) of cells in the M5-treated group and the control group at different time points. **(B-F)** qPCR validation of the relative expression levels of hub genes in the psoriasis cell model treated with M5. **(G-I)** qPCR analysis of inflammatory cytokines IL-6, IL-8, and TNF-α expression levels in the CD cell model stimulated with LPS. **(J-N)** qPCR validation of the relative expression levels of hub genes in the CD cell model stimulated with LPS. Statistical significance: ns indicates no significance, *P < 0.05, **P < 0.01, ***P < 0.001. Data are presented as mean ± SEM.

To validate the findings from transcriptomic, machine learning, and single-cell analyses, functional assays were performed using CCK-8 and RT-qPCR techniques to assess the expression of shared biomarkers. The CCK-8 assay demonstrated that M5-induced *in vitro* psoriasis models significantly enhanced the proliferation of HaCaT keratinocytes compared to controls (**Figure 10A**). Furthermore, RT-qPCR analysis showed significant upregulation of the shared biomarkers, including CCNB1, CEP55, DLGAP5, KIF4A, and NCAPG, in M5-treated HaCaT cells relative to the control group (**Figures 10B–F**). Among these, CCNB1 exhibited the most pronounced upregulation, with expression levels increasing 1.45-fold compared to controls. Similarly, in an *in vitro* inflammatory model of CD established by LPS (lipopolysaccharide) stimulation of HT-29 cells, RT-qPCR results revealed substantial increases in the mRNA levels of pro-inflammatory cytokines IL-6, IL-8, and TNF-α compared to the control group (**Figures 10G–I**). Concurrently, the shared biomarkers CCNB1, CEP55, DLGAP5, KIF4A, and NCAPG were also significantly upregulated (**Figures 10J–N**), with KIF4A exhibiting the greatest increase, reaching a 2.53-fold elevation relative to controls. In summary, these *in vitro* validation experiments for psoriasis and CD models validated the dysregulated expression of shared biomarkers during disease progression and reinforced the reliability of the integrative bioinformatics analyses. These findings lay a robust experimental foundation for further investigations into the molecular mechanisms underlying the roles of core genes in psoriasis and CD.

### 3.11 Identification of candidate drugs targeting hub genes in psoriasis and CD

**Figure 11:**
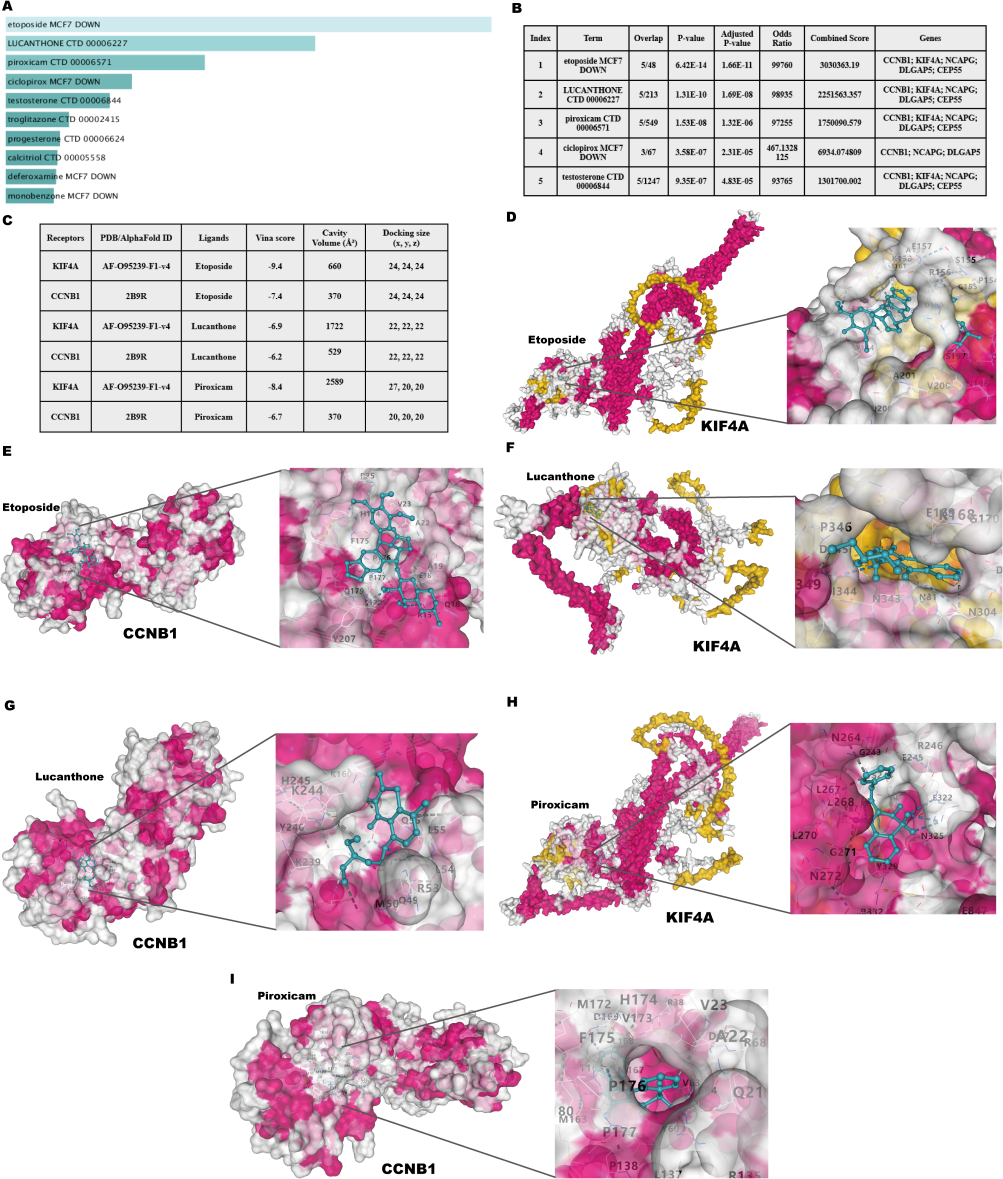
Identification of candidate drugs and molecular docking analysis targeting hub genes in psoriasis and CD. **(A)** Summary of candidate drugs identified from the DSigDB database. **(B)** Enrichment analysis results for candidate drugs targeting hub genes. **(C)** Docking scores and ligand-receptor interaction details for hub genes. **(D, E)** Molecular docking visualization of Etoposide with KIF4A and CCNB1. **(F, G)** Molecular docking visualization of Lucanthone with KIF4A and CCNB1. **(H, I)** Molecular docking visualization of Piroxicam with KIF4A and CCNB1.

Potential therapeutic drugs targeting the identified hub genes were systematically screened by analyzing p-values and binding scores. After multiple testing correction, several drugs, including Etoposide, Lucanthone, Piroxicam, and Ciclopirox, were identified as strong candidates based on their binding affinities and significant correlations with the hub genes (**Figure 11A**). Notably, these small-molecule compounds exhibited potential applicability as co-treatments for psoriasis and CD. Among the identified compounds, the analysis prioritized the top five candidates with promising therapeutic potential (**Figure 11B**). Collectively, these findings offer a foundation for experimental validation of these compounds as dual treatments for these diseases.

### 3.12 Molecular docking of candidate targets and related ingredients

Molecular docking analysis was performed to investigate the interactions between the two most significantly upregulated shared hub genes identified by RT-qPCR (CCNB1 and KIF4A) and the top three predicted candidate drugs (**Figure 11C**). A binding energy below 0 kcal/mol indicates docking activity, while a binding energy below −6 kcal/mol suggests favorable docking affinity. The analysis revealed that Etoposide exhibited the lowest binding energies with both CCNB1 (−7.9 kcal/mol) and KIF4A (−9.4 kcal/mol) among the tested compounds, indicating highly stable binding conformations. The visualized docking conformations provided additional evidence for these findings (**Figures 11D–I**). These results position Etoposide as the most promising candidate for the dual treatment of psoriasis and CD. As an approved chemotherapeutic agent, Etoposide has a long clinical history and well-established management protocols for its side effects. Through the strategy of drug repurposing, the immunosuppressive effects of Etoposide have been reassessed, offering potential new solutions for the treatment of psoriasis and CD. The therapeutic potential of Etoposide warrants further experimental validation and mechanistic investigations.

### 3.13 Validation of the therapeutic effects of Etoposide on HaCaT and HT-29 cell lines

**Figure 12:**
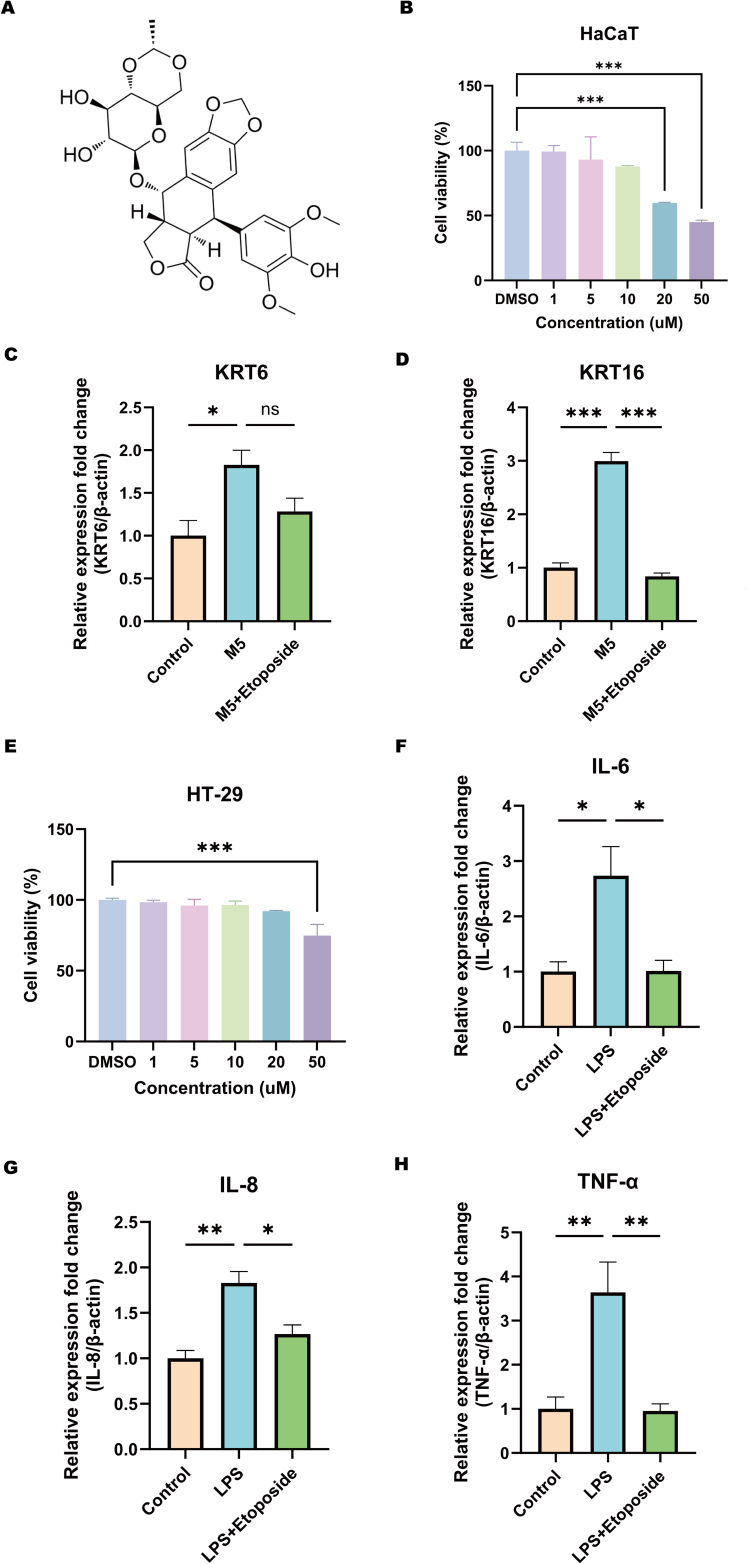
Effects of Etoposide on HaCaT and HT-29 cell lines. **(A)** Chemical structure of Etoposide. **(B)** CCK-8 assay showing the effect of different concentrations of Etoposide (1, 5, 10, 20, and 50 μM) on HaCaT cell viability. **(C, D)** qRT-PCR analysis of the expression levels of psoriasis-related marker genes KRT6 and KRT16 in HaCaT cells. HaCaT keratinocytes were treated with or without 1 μM Etoposide for 24 hours and simultaneously stimulated with M5 cocktail cytokines (10 ng/ml) or not for 24 hours. Cells were harvested, and RNA was extracted for qRT-PCR analysis, with β-actin as an internal reference. The following groups were included: DMSO (Control), DMSO+M5 (M5), M5+Etoposide (M5+Etoposide). **(E)** CCK-8 assay showing the effect of different concentrations of Etoposide (1, 5, 10, 20, and 50 μM) on HT-29 cell viability. **(F-H)** qRT-PCR analysis of the expression levels of CD-related inflammatory cytokines IL6, IL8, and TNF-α in HT-29 cells. HT-29 cells were treated with or without 1 μM Etoposide for 24 hours and simultaneously stimulated with LPS (20 μg/ml) or not for 24 hours. Cells were harvested, and RNA was extracted for qRT-PCR analysis, with β-actin as an internal reference. The following groups were included: DMSO (Control), DMSO+LPS (LPS), LPS+Etoposide (LPS+Etoposide). *P < 0.05, **P < 0.01, ***P < 0.001.

To further evaluate the therapeutic effects of Etoposide on HaCaT and HT-29 cell lines (**Figure 12A**), the impact of Etoposide on cell viability was first assessed using the CCK-8 assay. HaCaT cells were treated with Etoposide at concentrations of 1, 5, 10, 20, and 50 μM for 24 hours. The results demonstrated a dose-dependent cytotoxic effect of Etoposide, with concentrations above 20 μM significantly reducing cell viability (**Figure 12B**). Consequently, 1 μM was selected for further experiments with HaCaT cells. HT-29 cells were treated with the same concentration gradient of Etoposide for 24 hours, and concentrations above 50 μM significantly reduced cell viability (**Figure 12E**). Therefore, 1 μM was also chosen as the treatment concentration for subsequent experiments with HT-29 cells. Subsequently, HaCaT cells were stimulated with M5, and HT-29 cells were treated with LPS. The cells were then divided into the following groups: control, stimulation group, and Etoposide treatment group. Total RNA was extracted and analyzed using qRT-PCR to assess the expression levels of psoriasis-related keratinocytes marker genes (KRT6, KRT16) (**Figures 12C, D**) and CD-related inflammatory cytokines (IL6, IL8, TNF-α) (**Figures 12F–H**). The results showed that M5 and LPS stimulation significantly upregulated the expression of these genes in HaCaT and HT-29 cells, indicating a pro-inflammatory and hyperproliferative state. However, Etoposide treatment effectively reversed the induced gene upregulation, significantly reducing the expression levels of KRT6, KRT16, IL6, IL8, and TNF-α.

These findings validate the therapeutic potential of Etoposide in modulating inflammatory responses and inhibiting abnormal keratinocytes activation. This observation aligns with molecular docking predictions, suggesting that Etoposide may exert protective effects in inflammatory skin and intestinal diseases. Future studies will further investigate the specific signaling pathways involved in its action.

## 4 Discussion

Psoriasis and CD are chronic inflammatory conditions characterized by immune dysregulation, genetic predisposition, and cyclical flare-ups (10, 11). Both diseases share overlapping genetic and environmental factors, as demonstrated by epidemiological studies. For instance, a nationwide Danish cohort of over 5.5 million adults found a strong correlation between the two (57). In addition, a meta-analysis of 93 studies reported a psoriasis prevalence of 3.6% in CD patients and 2.8% in ulcerative colitis (UC) patients, with higher rates among pediatric CD cases. Individuals with psoriasis were shown to have a 1.7-fold increased risk of developing CD, highlighting a significant bidirectional association (12). Similar findings have been reported in other studies (13). Furthermore, genetic evidence further supports the link between these diseases. A Mendelian randomization study using GWAS data from 463,372 individuals of European ancestry demonstrated a significant increase in risk associated with genetic predisposition to inflammatory bowel disease (IBD), particularly in CD patients (58). However, the reverse relationship remains unclear, indicating a need for further elucidation of the underlying genetic and molecular mechanisms. In summary, psoriasis and CD exhibit robust associations across epidemiological, genetic, and immunological domains (10, 14–17). While existing research has primarily focused on epidemiological aspects, further exploration into the molecular mechanisms is necessary to identify key biomarkers and therapeutic targets. This study aims to fill this knowledge gap through the use of bioinformatics to analyze bulk transcriptomic and single-cell sequencing data, identifying shared biomarkers and regulatory pathways. Molecular experiments and docking analyses will validate potential therapeutic targets to provide insights into common mechanisms and treatment strategies for these diseases.

Using WGCNA and differential analysis, 79 shared genes were identified as being implicated in psoriasis and CD, with functional enrichment demonstrating a strong association with cell cycle processes. KEGG and GSEA analyses revealed significant enrichment in pathways associated with cell division and inflammation, including E2F targets, G2M checkpoints, MYC targets, interferon-γ response, TNF-α signaling via NF-κB, and IL-6/JAK/STAT3 signaling. These results indicate that immune dysregulation and abnormal cell proliferation serve as common drivers of disease progression, highlighting potential therapeutic targets.

Machine learning identified five hub genes—KIF4A, DLGAP5, NCAPG, CCNB1, and CEP55—as shared molecular biomarkers. These genes are essential for cell cycle regulation, including processes such as chromosome separation, spindle assembly, and regulation of the G2/M transition.

KIF4A (Kinesin Family Member 4A) is a microtubule-associated motor protein involved in the regulation of chromosome condensation and separation during mitosis. Studies have shown that KIF4A is overexpressed in various cancers and contributes to tumorigenesis by influencing cell proliferation, migration, and the tumor microenvironment (59–64). However, research on the role of KIF4A in psoriasis and CD remains relatively scarce. Based on the existing findings, we hypothesize that KIF4A may regulate cell proliferation by maintaining mitosis, contributing to the abnormal proliferation of keratinocytes and intestinal epithelial cells in the pathogenesis of psoriasis and CD, thereby interfering with tissue repair processes.

DLGAP5 (Discs Large Homolog Associated Protein 5) is a microtubule-associated protein that plays a critical role in mitotic spindle assembly and stabilization. It influences cell proliferation and migration, particularly in various cancers (65–67), though reports on its involvement in psoriasis and CD are limited. Research by Yujie Li has shown that DLGAP5 regulates breast cancer cell proliferation, migration, invasion, and the cell cycle via the JAK2/STAT3 signaling axis (68). Therefore, upregulation of DLGAP5 may affect the abnormal differentiation of keratinocytes in psoriasis and intestinal epithelial cell repair in CD by enhancing cell proliferation and mitosis regulation.

NCAPG (Non-SMC Condensin I Complex Subunit G) is essential for chromosome condensation and stability during mitosis. Its overexpression has been linked to tumorigenesis, promoting cell proliferation, invasion, migration, and resistance to apoptosis (69, 70). Specifically, Ding-Ping Sun’s research revealed that NCAPG is highly expressed in colorectal cancer (CRC) tissues (71), and its downregulation inhibits CRC cell proliferation, migration, and invasion by interfering with the G2/M to G1 cell cycle transition (72). Moreover, NCAPG has been identified as a susceptibility gene for psoriasis (73), underscoring its role in both diseases and justifying its investigation as a key gene in cell cycle regulation.

CCNB1 (Cyclin B1) is a key regulatory factor in cell cycle progression, particularly in the G2/M transition (54). Abnormal expression of CCNB1 has been observed in various cancers and inflammatory diseases (54). In psoriasis, CCNB1 is closely associated with cell cycle regulation, highlighting its role in controlling keratinocytes proliferation (74). Additionally, studies on celiac disease have shown that CCNB1 is highly expressed in epithelial cells of affected patients, correlating with increased cell proliferation and mitosis, which overlaps with features of CD (75). Therefore, abnormal expression of CCNB1 may accelerate the cell cycle, contributing to excessive proliferation of keratinocytes in psoriasis and impaired repair of intestinal epithelial cells in CD.

CEP55 (Centrosomal Protein 55) is implicated in mitosis and the regulation of the PI3K/AKT pathway. Its dysregulation is associated with various cancers and inflammatory diseases (76). Although research on its role in CD is limited, CEP55 has been identified as a key gene associated with immune responses, cell cycle regulation, and the Wnt signaling pathway in psoriasis (74).

Immune cell infiltration analysis revealed significant involvement of 12 immune cell types, including activated CD4+ T cells, γδ T cells, Th17 cells, and regulatory T cells (Tregs). CD4+ T cells were pivotal in both diseases, promoting keratinocytes proliferation in psoriasis and exacerbating intestinal inflammation in CD through cytokine secretion. Dysregulation of Tregs and Th17 cells further amplifies inflammation, highlighting the immune-mediated mechanisms common to both diseases.

Specifically, in psoriasis, the early infiltration of activated CD4^+^ T cells into the epidermis acts as a key trigger for inflammation and keratinocytes hyperproliferation (77, 78). Activated CD4+ T cells can differentiate into Th17 cells, which secrete pro-inflammatory cytokines such as IL-17A, IL-17F, and IL-22, directly promoting keratinocytes proliferation and inflammation (79, 80). Additionally, γδ T cells contribute to psoriasis by secreting pro-inflammatory cytokines such as IL-17A, IL-17F, and IL-22, promoting inflammation and keratinocytes proliferation (81–83).

Similarly, in CD, T cell dysregulation, particularly the imbalance between Th17 cells and Tregs, is a hallmark of intestinal inflammation (84). The therapeutic potential of Treg cells in CD has been widely validated (85). The reduction in Treg cell function exacerbates immune responses, while an increase in Th17 cells leads to enhanced secretion of pro-inflammatory cytokines. Recent studies have shown that although regulatory T cell (Treg) infiltration is elevated in both psoriasis and CD, their suppressive function is often impaired due to the influence of the inflammatory microenvironment. In psoriasis, despite an increased number of Tregs in lesional skin, their anti-inflammatory capacity is compromised, potentially due to cytokine-induced functional dysregulation (86). Similarly, in CD, Tregs accumulate in inflamed intestinal mucosa but fail to effectively restore immune homeostasis due to functional impairments (85). This paradox of increased Treg infiltration alongside persistent inflammation suggests that Treg expansion may represent a compensatory response by the immune system to counteract chronic immune activation. However, the inflammatory milieu likely disrupts their regulatory function, rendering them unable to effectively suppress pathogenic immune responses (87).

Currently, while biologic therapies targeting TNF-α and IL-12/23 have demonstrated efficacy in treating both conditions (88, 89), paradoxical effects, such as psoriasis exacerbation in IBD patients (89–93), highlight the need for alternative therapeutic approaches. Using the DSigDB database, we identified potential small-molecule drugs, including Etoposide, Lucanthone, and Piroxicam, capable of modulating cell cycle regulation and inflammatory pathways. Etoposide, a topoisomerase inhibitor, induces cell cycle arrest and apoptosis (94), while Lucanthone and Piroxicam exhibit anti-inflammatory and immunomodulatory properties, and represent promising therapeutic candidates for further investigation. Molecular docking simulations revealed that Etoposide exhibited the strongest binding affinity to core targets, significantly outperforming Lucanthone and Piroxicam. As an FDA-approved chemotherapeutic agent, Etoposide benefits from a well-established manufacturing process and demonstrates high cost-effectiveness. Etoposide may provide a viable alternative therapeutic option in certain patients, especially those who develop resistance or inadequate responses to biologics. However, given its potential long-term side effects, careful risk-benefit assessment is warranted in clinical applications.

Our study identified shared biomarkers and pathways between psoriasis and CD, highlighting the critical roles of cell cycle dysregulation and immune responses. Additionally, we preliminarily screened potential therapeutic agents. These findings lay the foundation for novel therapeutic strategies while also underscoring several limitations. Reliance on public datasets may contribute to variability, highlighting the need for further molecular and clinical validation of the regulatory mechanisms underlying the identified biomarkers. Future research should focus on elucidating the intricate interactions among hub genes and immune pathways to advance our understanding of these interconnected diseases.

## 5 Conclusion

By integrating bioinformatics, machine learning, and molecular validation, this study demonstrates that cell cycle regulation, immune dysregulation, and inflammation represent critical shared pathogenic mechanisms between psoriasis and CD. Five novel shared biomarkers—KIF4A, DLGAP5, NCAPG, CCNB1, and CEP55—were identified, that play significant roles in cell cycle-related processes and exhibit close associations with CD4+ T cells and γδ T cells. Additionally, this study identified Etoposide, Lucanthone, and Piroxicam as potential therapeutic candidates targeting these biomarkers. These findings not only offer valuable insights into the development of therapeutic strategies for psoriasis and CD but also highlight the potential clinical diagnostic and therapeutic applications of the identified genes.

## Data Availability

The datasets presented in this study can be found in online repositories. The names of the repository/repositories and accession number(s) can be found in the article/**Supplementary Material**.
