# Supplementary material for: Identification and validation of shared biomarkers and drug repurposing in psoriasis and Crohn’s disease: integrating bioinformatics, machine learning, and experimental approaches
Source: Front Immunol. 2025 May 8;16:1587705. doi: 10.3389/fimmu.2025.1587705 (PMC12095375; doi:10.3389/fimmu.2025.1587705)
Supplement: Supplementary file 1 [file DataSheet1.docx]

**223 overlapping DEGs in psoriasis and CD:**

PI3、S100A12、TCN1、S100A9、PDZK1IP1、ZC3H12A、CXCR2、GNA15、MELK、RRM2、MPZL2、CDC20、CCL20、LCN2、NMI、CD24、CDH3、DLGAP5、SLC6A14、CARD6、FOXM1、STAT1、CKS2、PNP、IFI30、WNT5A、KIF20A、NFKBIZ、S100A11、UBE2T、LAMP3、PSMB8、GGH、COBL、APOL1、CDKN3、NUSAP1、IFI16、PBK、PHYH、NAMPT、HK2、ABCC1、PRDM1、CEP55、NCAPG、SLC7A5、CCNB1、KPNA2、SLPI、LAP3、PRC1、EHF、KIF4A、TGFBI、TPX2、PYGL、TMEM116、CXCL1、TLE1、TTK、CHP2、CD274、MMP12、LRG1、CCNA2、CCND1、VSNL1、CDC6、IDO1、TRIM16、CDC25B、SLC7A11、SHCBP1、GBP2、SMPD3、SLC38A5、TRIM22、MATN2、KIF11、CENPE、CDHR1、GINS1、FAM234B、NETO2、GBP5、RND1、STIL、SKA3、PRLR、TRIB2、CXCL9、OSR2、CLDN8、LYN、PFN2、CXCL10、IRF1、HCAR3、KIF14、GLT1D1、AQP9、SLAMF7、SPART、CXCL8、P2RY2、HMMR、LIPG、HLF、CENPK、SULT2B1、IL7R、PSMB9、ANLN、MLKL、CTSL、PLEK、LYPD1、BATF2、CRIP1、SCIN、OAS2、MAOA、CYP2J2、MKI67、FOXD1、SLITRK6、SGO2、NOS2、MT1X、FRMD3、IFITM1、RAB31、PARP9、ANKH、TNIP3、GREM1、PLAU、C2orf88、SUSD2、CYP2S1、ALDOC、ANKRD22、SEMA3G、CLDN1、XKRX、IL1B、FZD7、EPHX1、SELE、CCL2、EPSTI1、CMBL、CNTN4、NFE2L3、ACOX2、DEPDC1、NCF2、RAB23、CCL8、LYPD6B、IGFBP5、IL20RA、SORD、MMP1、DOCK4、GPAT3、PMP22、IFI44、ASPM、LY96、GNG11、COL1A2、ASPA、FERMT2、LYZ、SLC46A3、PTGR1、SLC28A3、LGALS2、PAG1、MUC4、ACKR4、ALDH1A2、PLCB1、SLC26A2、CFH、LGR5、SOD2、F10、HSD17B2、FADS1、ROR1、S100P、EMCN、GRAMD1C、HSD11B2、CXCL11、CCR1、COL3A1、TBX3、HSD3B1、RBP4、SERPINA3、C7、BST2、ACSL1、RGS13、TPPP3、KCNK5、FMO1、CREB3L1、LPL、MUC1、CEACAM6、ZG16B、CEACAM7、SLC26A3、COL12A1、AGT、FBP1、AGR2、MMP3

**Table S1 PCR primer sequences.**

| RNA | Forward | Reverse |
| --- | --- | --- |
| CCNB1  CEP55  DLGAP5  KIF4A  NCAPG  IL-6  IL-8  TNF-α  β-actin  KRT6  KRT16 | 5′-AACATCTGGATGTGCCCCTG-3′  5′-GGAGGGAGCAGGTGTTGAAAGC-3′  5′-CGCACAGCAGTTGGTCAAACAAG-3′  5′-AACGCCATCTGAATGACCTCCTTG-3′  5′-CAGCACAGGATGCCACCTTGAC -3′  5′-ACTCACCTCTTCAGAACGAATTG-3′  5′-ACTGAGAGTGATTGAGAGTGGAC-3′  5′-CCTCTCTCTAATCAGCCCTCTG-3′  5′-ATAGCACAGCCTGGATAGCAA-3′  5′-GGGTTTCAGTGCCAACTCAG-3′  5′-TTATTAGCCCACCACCAGCAG-3′ | 5′-GGTCTCCTGCAACAACCTGA-3′  5′-TGAAGCAGTTTGGAGCCACAGTC -3′  5′-TGCCACCCAGATTCCTCAAGTTTG-3′  5′-TCCGAAACTTGACCACGCACTTC -3′  5′-CTTTCAGAGTCGGCTTCAGCAGTC -3′  5′-CCATCTTTGGAAGGTTCAGGTTG-3′  5′-AACCCTCTGCACCCAGTTTTC-3′  5′-GAGGACCTGGGAGTAGATGAG-3′  5′-AATCTGGCACCACACCTTCTA-3′  5′-CCAGGCCATACAGACTGCGG-3′  5′-TTATTAGCCCACCACCAGCAG-3′ |

**
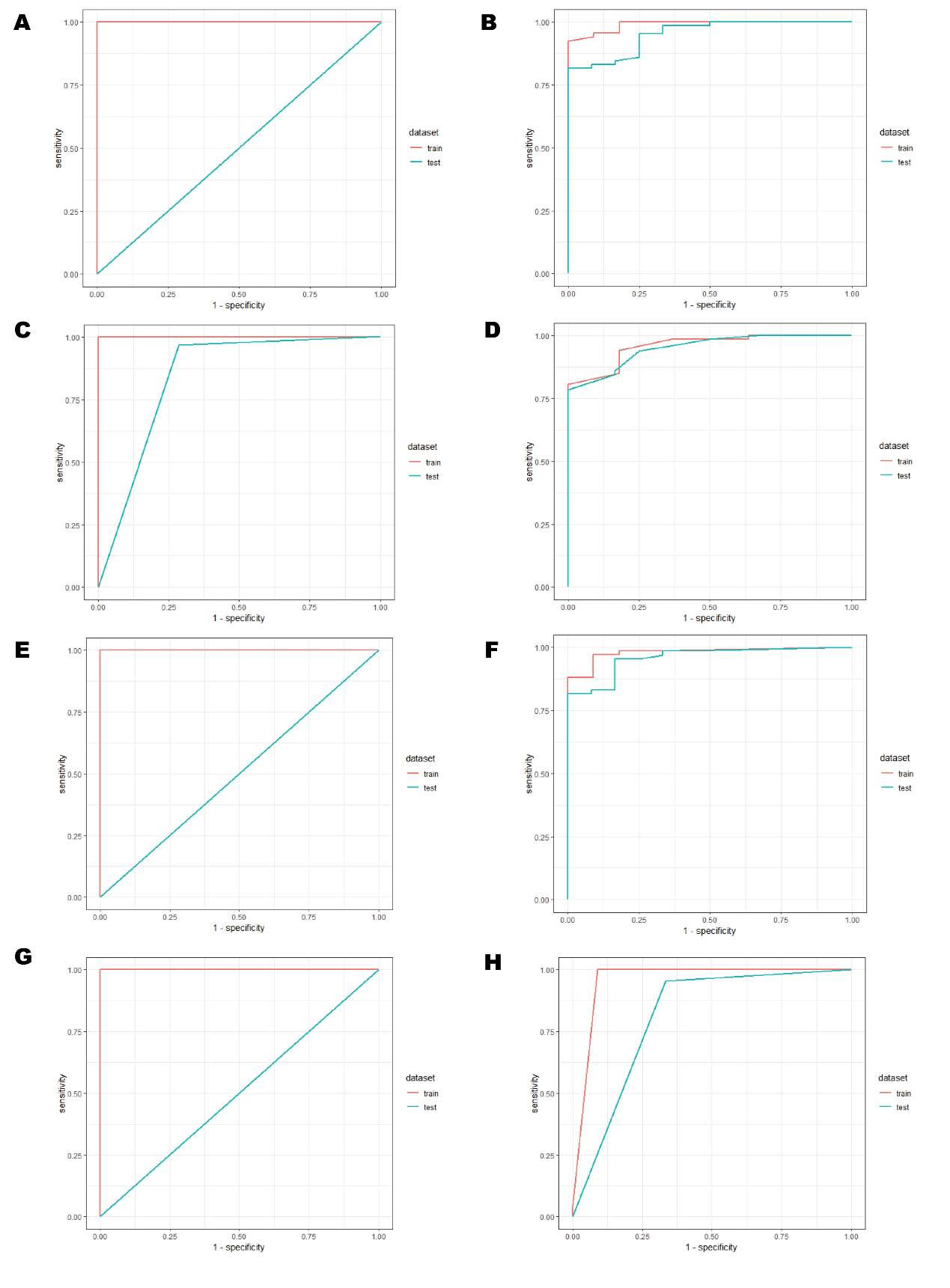
**

**Supplementary Figure 1** (A, B) ROC curves of the RF model for psoriasis and CD on training and testing sets. (C, D) ROC curves of the KNN model for psoriasis and CD on training and testing sets. (E, F) ROC curves of the XGBoost model for psoriasis and CD on training and testing sets. (G, H) ROC curves of the Dtree model for psoriasis and CD on training and testing sets.

**
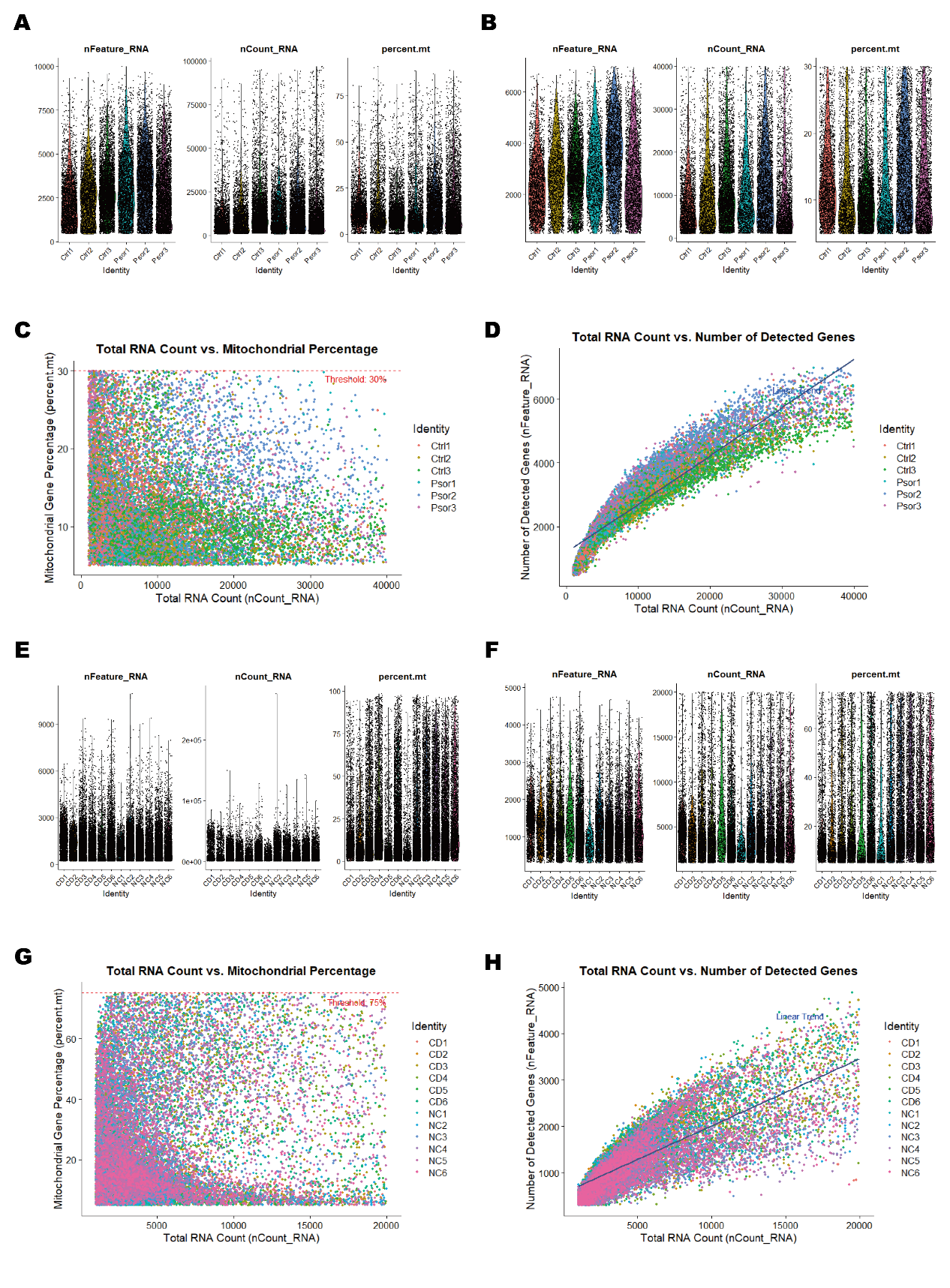
**

**Supplementary Figure 2** (A, B) Violin plots showing quality control metrics, including detected genes, UMI counts, and mitochondrial gene percentages, for cells in the psoriasis dataset (GSE162183) before and after filtering. (C) Scatter plot of UMI counts versus detected genes in the psoriasis dataset, with filtering thresholds shown by dashed lines. (D) Scatter plot of UMI counts versus mitochondrial gene expression percentage in the psoriasis dataset, with thresholds indicated by dashed lines. (E, F) Violin plots showing quality control metrics for cells in the CD dataset (GSE214695) before and after filtering. (G) Scatter plot of UMI counts versus detected genes in the CD dataset. (H) Scatter plot of UMI counts versus mitochondrial gene expression percentage in the CD dataset.

**
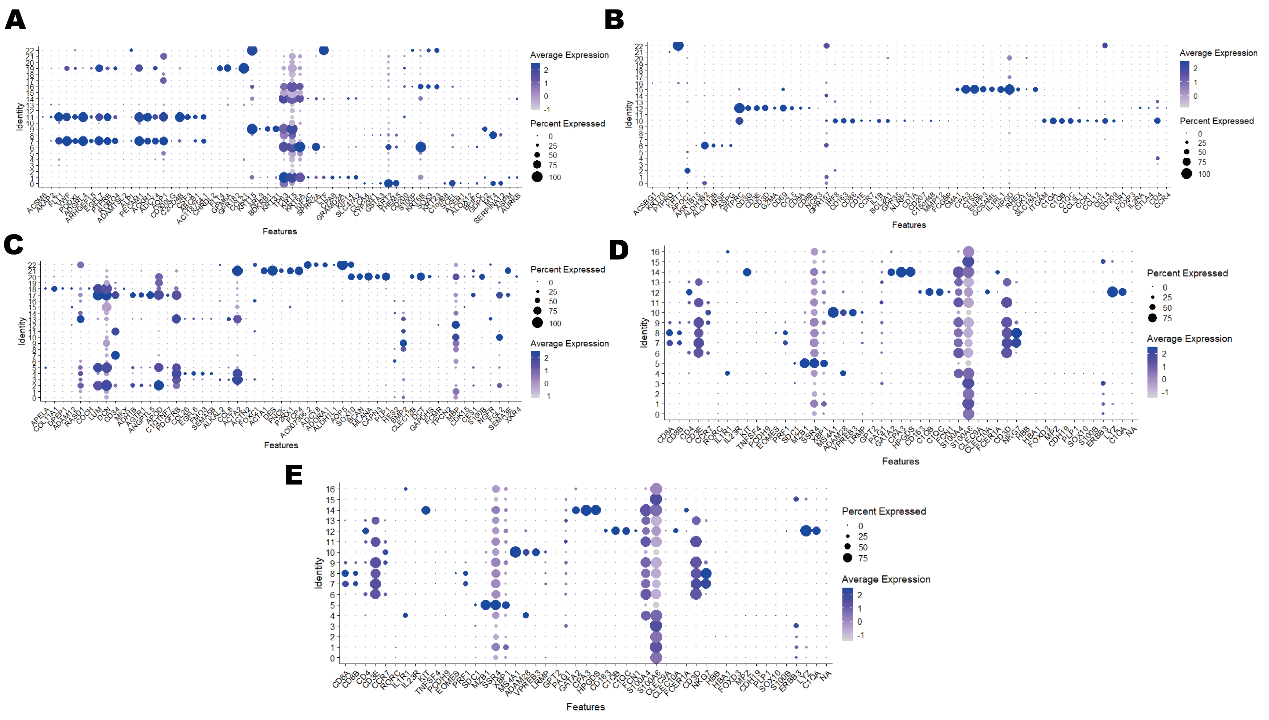
**

**Supplementary Figure 3** (A-C) Annotation of cell clusters in the psoriasis dataset (GSE162183), with a dot plot showing the expression levels of key marker genes across different cell types. (D, E) Annotation of cell clusters in the CD dataset (GSE214695), with a dot plot showing the expression levels of key marker genes across different cell types.
